# Regulating a Post-Transcriptional Regulator: Protein Phosphorylation, Degradation and Translational Blockage in Control of the Trypanosome Stress-Response RNA-Binding Protein ZC3H11

**DOI:** 10.1371/journal.ppat.1005514

**Published:** 2016-03-22

**Authors:** Igor Minia, Christine Clayton

**Affiliations:** Zentrum für Molekulare Biologie der Universität Heidelberg (ZMBH), DKFZ-ZMBH Alliance, Heidelberg, Germany; University of Dundee, UNITED KINGDOM

## Abstract

The life cycle of the mammalian pathogen *Trypanosoma brucei* involves commuting between two markedly different environments: the homeothermic mammalian host and the poikilothermic invertebrate vector. The ability to resist temperature and other stresses is essential for trypanosome survival. Trypanosome gene expression is mainly post-transcriptional, but must nevertheless be adjusted in response to environmental cues, including host-specific physical and chemical stresses. We investigate here the control of ZC3H11, a CCCH zinc finger protein which stabilizes stress response mRNAs. ZC3H11 protein levels increase at least 10-fold when trypanosomes are stressed by heat shock, proteasome inhibitors, ethanol, arsenite, and low doses of puromycin, but not by various other stresses. We found that increases in protein stability and translation efficiency both contribute to ZC3H11 accumulation. ZC3H11 is an *in vitro* substrate for casein kinase 1 isoform 2 (CK1.2), and results from CK1.2 depletion and other experiments suggest that phosphorylation of ZC3H11 can promote its instability *in vivo*. Results from sucrose density centrifugation indicate that under normal culture conditions translation initiation on the *ZC3H11* mRNA is repressed, but after suitable stresses the *ZC3H11* mRNA moves to heavy polysomes. The *ZC3H11* 3'-UTR is sufficient for translation suppression and a region of 71 nucleotides is required for the regulation. Since the control works in both bloodstream forms, where *ZC3H11* translation is repressed at 37°C, and in procyclic forms, where *ZC3H11* translation is activated at 37°C, we predict that this regulatory RNA sequence is targeted by repressive *trans* acting factor that is released upon stress.

## Author Summary

Like other organisms, the mammalian pathogen *Trypanosoma brucei* is able to sense environmental changes and to change its gene expression accordingly. In contrast with other organisms, however, trypanosomes and related kinetoplastids effect these changes almost exclusively by controlling the translation of mRNAs into protein, and by adjusting the rate at which the mRNAs are degraded. ZC3H11 is an RNA binding protein, which stabilizes mRNAs that encode chaperones. Chaperones are needed to refold proteins after stress. Under normal growth conditions ZC3H11 protein is very unstable, and in addition, not much of the protein is made. Although *ZC3H11* mRNA is present under normal, unstressed conditions, most of it is not translated. However, when the cells were stressed by elevated temperature, arsenite, ethanol, puromycin or proteasome inhibitors the amount of ZC3H11 rose almost 10-fold. This was caused by a combination of increased protein stability and enhanced translation of the mRNA. We found that a 71 nucleotide segment of the 3'-untranslated region of the *ZC3H11* mRNA was responsible for the regulated translational blockage. We also obtained evidence that casein kinase 1 isoform 2 might phosphorylate ZC3H11, and that phosphorylation can promote ZC3H11 protein degradation. Overall, our results show that the increase in the ZC3H11 level after stress occurs because of changes in protein synthesis, phosphorylation, and stability.

## Introduction

The African trypanosome *Trypanosoma brucei* is responsible for sleeping sickness in humans and nagana in livestock. Bloodstream-form trypanosomes, which are found in mammalian blood and tissue fluids, are exposed to temperatures ranging from about 36°C to 40°C (fever). Trypanosomes are transmitted by Tsetse flies, where they replicate as procyclic forms in the midgut, progressing to epimastigotes, then metacyclic forms in the salivary glands. Within Tsetse, the temperature may fluctuate between 20°C and 43°C [1]. In addition, availability of nutrients in the two hosts is different. Trypanosomes, like other kinetoplastids, manage these changes almost exclusively through post-transcriptional mechanisms. Transcription is polycistronic and individual mRNAs are generated by processing: this precludes transcription control at the level of individual open reading frames. In contrast, there is extensive evidence for regulation of mRNA stability [2] and translation [3,4]. This regulation is often determined by sequences in the 3'-untranslated regions (3'-UTRs) of mRNAs, and mediated by RNA binding proteins [5–8].

It has long been known that kinetoplastids, like other organisms, respond to temperature stress by inducing synthesis of heat shock proteins (HSPs), and shutting down synthesis of other proteins. The response of *T*. *brucei* to heat stress includes repression of transcription [9], mRNA processing [10–12] and translation [13]. Heat shock and other stresses also cause the formation of stress granules that contain translationally silenced mRNAs [13,14]. Several studies in kinetoplastids have demonstrated roles of 3'-UTRs in determining the stability and translation of HSP mRNAs [13,15–19]. We recently found an RNA binding protein called ZC3H11, which is required for preferential retention of the transcripts upon heat shock and for survival of the parasites after heat shock [20]. This protein binds to mRNAs encoding major cytoplasmic HSPs including HSP70, HSP83, HSP100, HSP110, HSP20, DNAJ1, DNAJ2, and FKBP. Each of these mRNAs contains multiple repeats of the AU-rich element (UAU)_n_ within their 3'-UTRs. These repeats are bound by ZC3H11 and required for the response of the mRNA to heat shock [20]. ZC3H11 can act through recruitment of a complex containing three essential proteins: MKT1, LSM12 and PBP1, which in turn recruit poly(A) binding proteins (PABP) to the 3'-UTR, with consequent mRNA stabilization [21].

ZC3H11 is almost undetectable in trypanosomes grown at normal temperature, whether they are bloodstream forms at 37°C or procyclic forms at 27°C. Upon heat shock, however, ZC3H11 protein becomes readily detectable. It migrates much slower than expected on denaturing polyacrylamide gels, which is partially due to heavy phosphorylation [20]. In this paper, we aimed to understand the mechanism by which the abundance of ZC3H11 increases after heat shock. We present evidence for changes in mRNA translation, protein modification and protein stability, and identify a region within the 3'-UTR of the *ZC3H11* mRNA that is responsible for heat-regulated translation.

## Results

### ZC3H11 expression is increased by treatments that cause accumulation of incompletely folded proteins in the cytosol

**Fig 1:**
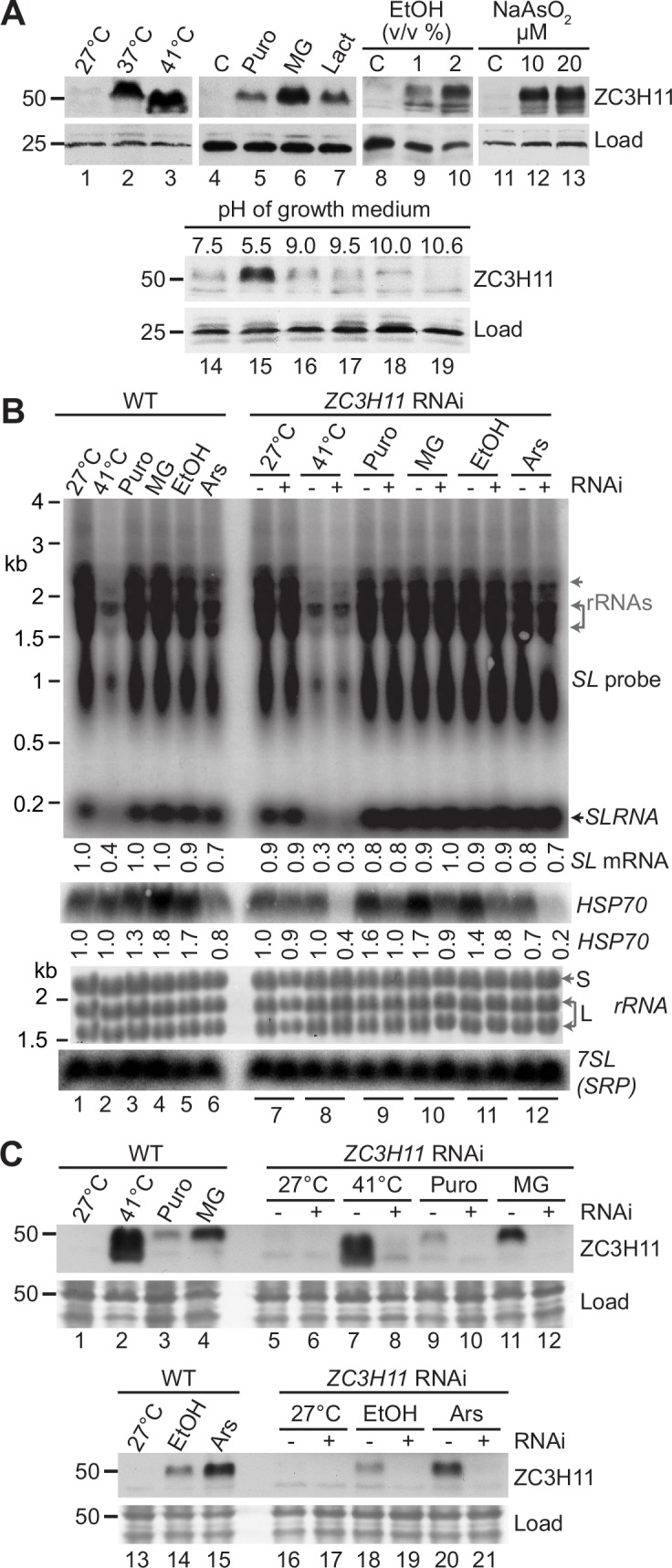
ZC3H11 increases after treatments that cause accumulation of misfolded proteins in the cytosol. **A.** Expression of ZC3H11 in procyclic cells under different conditions. Cells were treated for one hour each with mild (37°C) or severe (41°C) heat shock, puromycin (Puro, 1 μg/ml), MG132 (MG, 10 μg/ml), lactacystin (Lact, 10μM), ethanol, sodium arsenite, or altered pH. Cytoskeleton-free extracts from 5×10^6^ cells were loaded onto each lane, and ZC3H11 was detected on Western blots using anti-ZC3H11 antibodies. A cross-reacting band served as loading control ("Load"). **B.** Procyclic trypanosomes were subjected to stresses as in (A). We used either wild-type cells (WT, lanes 1–6) or cells with inducible RNAi targeting ZC3H11 (lane pairs 7–12). To induce RNAi, tetracycline (200ng/ml) was added 2 days previously. RNA was prepared and Northern blots were hybridised with probes for the spliced leader RNA (SL) to detect total mRNA and the spliced leader precursor RNA (SLRNA); for HSP70 (the Lister 427 homologue of Tb927.11.11330) and for the 7SL (signal recognition particle) RNA as loading control, with the methylene blue stained rRNA above. "S" indicates the major small subunit rRNA and "L" the two biggest two fragments of the large subunit rRNA. The positions of rRNA migration are also indicated on the SL-probed image. The banding pattern in this region is not due to cross-hybridisation with rRNA since rRNA is not reduced at 41°C. **C.** Effect of RNAi and stresses on levels of ZC3H11 protein, controls for panel B. Details are as in (A), except that the Ponceau S staining serves as the loading control.

To analyse expression of native ZC3H11, we made a polyclonal antiserum to the N-terminal 119 amino acids (S1A–S1D Fig). This antiserum specifically detects ZC3H11 in cytoskeleton-free detergent extracts (S1B Fig). Fig 1A shows native, untagged ZC3H11 expression after a variety of stresses. The predicted molecular weight of ZC3H11 is 40 kDa. Under normal culture conditions (lane 1), it is barely detectable. As expected, heat shock at 37°C resulted in the appearance of a band that migrated above 50 kDa (Fig 1, lane 2 and S1 Fig). In our previous experiments, we found that V5-tagged ZC3H11 (molecular weight about 42 kDa) migrated at about 60 kDa, and phosphatase treatment reduced this to about 50 kDa. The aberrantly slow migration of ZC3H11 is thus due partly to phosphorylation, and partly to some intrinsic characteristics of the protein sequence. Lane 3 shows the effect of a one-hour heat shock at 41°C, which is the temperature that was used in previous publications [13,20]. Despite the severity of this treatment, trypanosomes are able to resume RNA synthesis almost immediately upon returning to 27°C [13] and they also resume growth [20]. After the 41°C heat shock, the ZC3H11 band that was recognised by the antibody migrated faster in polyacrylamide gels; this was previously seen with the V5-tagged protein, and could be due either to dephosphorylation, or to protein degradation. By quantitative immunoblotting (S1E and S1F Fig) we estimated that procyclic cells grown at 27°C contain no more than 2×10^3^ ZC3H11 molecules per cell, increasing to 15–20×10^3^ molecules per cell at 37 or 41°C. As seen for *in situ* tagged protein [20], ZC3H11 levels were also increased by low doses of puromycin, and by the proteasome inhibitors MG132 and lactacystin [20] (Fig 1A, lanes 4–7).

In order to determine the specificity of ZC3H11 induction we subjected procyclic forms to variety of other stresses: ethanol, sodium arsenite, altered pH, and ER stress from dithiothreitol (which causes protein misfolding in the endoplasmic reticulum by reducing disulfide bonds) or tunicamycin (which causes accumulation of unfolded glycoproteins in the endoplasmic reticulum). We also tested two inhibitors of ribosome translocation, hygromycin and G418, and the effects of cold shock at 4°C and 16°C. Just three of these new stresses were found to increase the ZC3H11 levels: ethanol (Fig 1A, lanes 8–10), sodium arsenite (Fig 1A, lanes 11–13) and pH 5.5 (Fig 1A, lane 15). The response to acid, but not alkaline, pH makes sense since the Tsetse midgut has a pH range of about 8–10.5 [22]. Inhibitors of translation elongation (S2A Fig, lanes 1–12) and cold shock (S2A Fig, lanes 23–25) did not increase ZC3H11 levels. It was notable that the endoplasmic reticulum stress inducers also had no effect (S2A Fig, lanes 13–22). Endoplasmic reticulum stress is known to trigger a signal transduction cascade that leads to a shut-off of spliced leader transcription or "spliced leader silencing" [23,24]. The ZC3H11 stress response is clearly separate from this: instead, the common feature of the ZC3H11-inducers is that they are likely to cause cytosolic accumulation either of incompletely folded proteins, or, for puromycin, of protein fragments which may not be able to attain native conformations.

To find out whether *HSP70* mRNA levels are affected by the new stresses, and also whether such responses depend on ZC3H11, we tested cells with *ZC3H11* RNAi (Fig 1B and 1C). The amount of total mRNA can be assessed by hybridizing Northern blots with the spliced leader (*SL*) sequence that is found at the 5'-end of every trypanosome mRNA (Fig 1B). This gives a smear with two regions of more concentrated signal around about 2kb and 1kb. Heat shock inhibits transcription, so after an hour, there is a substantial decrease in the amount of total mRNA; but the *HSP70* mRNA escapes destruction (Fig 1B, lanes 1, 2 and 8). With the exception of arsenite (Fig 1B, lanes 6 and 12), the other stressors had no effect on total mRNA (Fig 1B, lanes 3–6), suggesting that they did not inhibiti transcription; all but arsenite caused slight increases in *HSP70* mRNA abundance (Fig 1B, lanes 3–5 and 9–11). RNAi targeting *ZC3H11* did not affect the abundance of *HSP70* mRNAs at 27°C (Fig 1B, lanes 7) but prevented its persistence at 41°C (Fig 1B, lanes 8) or after arsenite (Fig 1B, lanes 12). *ZC3H11* RNAi also prevented *HSP70* mRNA accumulation after puromycin, MG132 and ethanol treatment (Fig 1B, lanes 9–11). Heat shock was the only stress that led to an increase in ZC3H11 protein mobility (Fig 1A, lane 3, and 1C, lanes 2 and 7). Puromycin treatment in bloodstream forms also led to accumulation of only the slower-migrating form of ZC3H11 [20].

### Heat shock stabilizes ZC3H11 protein

**Fig 2:**
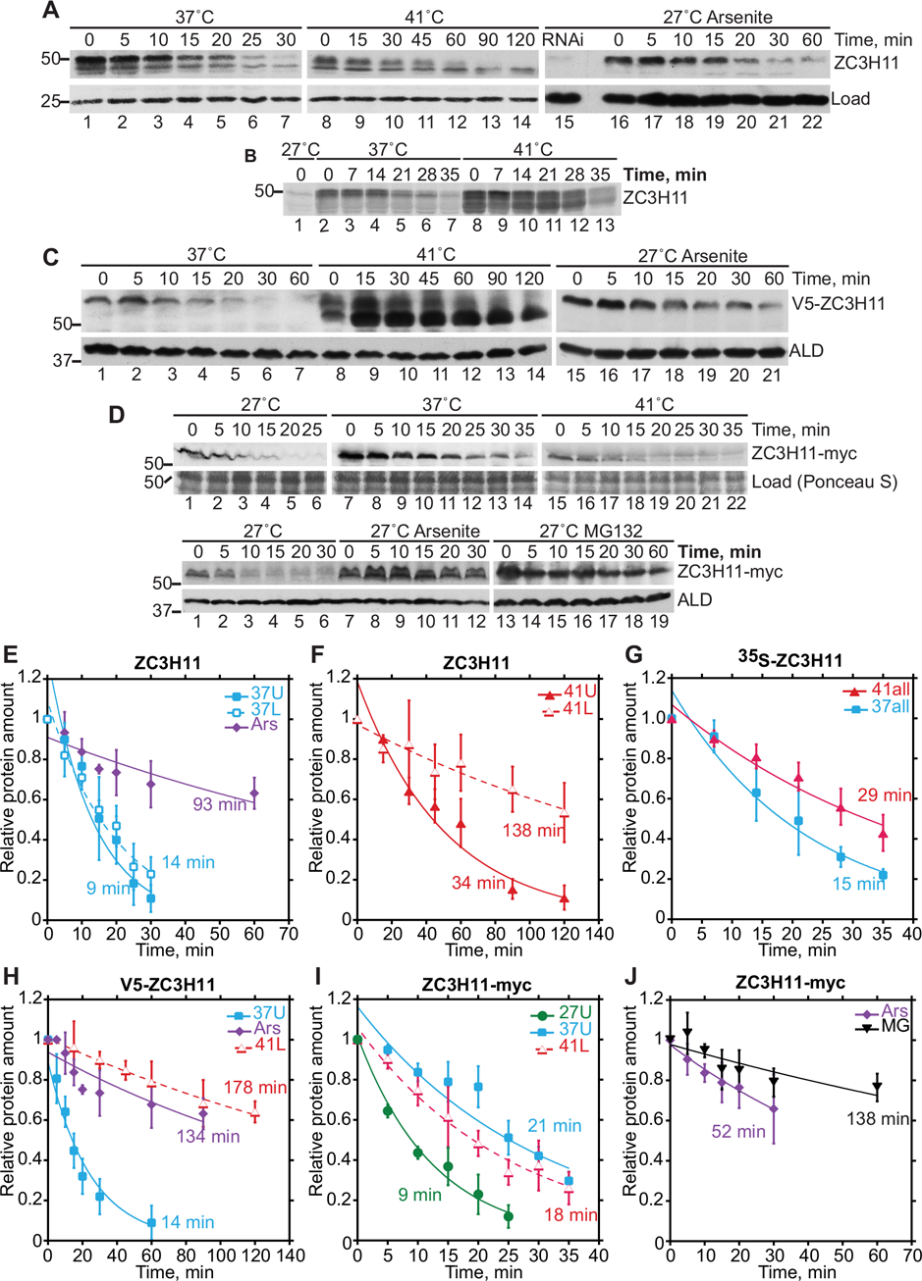
ZC3H11 protein becomes more stable upon heat shock. **A.** Half-life of ZC3H11 protein. Procyclic cells were either heat-shocked at 37°C (lanes 1–7) or 41°C (lanes 8–14), or incubated with 10μM sodium arsenite for 1 hour (lanes 15–22), then treated with cycloheximide (100μg/ml) to shutoff translation. Samples were analyzed by Western blotting. One representative blot is shown; a stable cross-reacting band migrating elsewhere on the gel is the loading control. The signals from the two bands were quantified independently by densitometry. Cells with RNAi (lane 15) serve as a control to show the lack of any cross-reacting proteins co-migrating with the "lower" ZC3H11 band. **B.** Half-life of ZC3H11 protein measured by [^35^S]-methionine pulse-chase. Procyclic cells were heat-shocked at 37°C or 41°C for 1h, incubated in methionine-free labelling medium for 15min, [^35^S]-methionine was added for 20min, then the cells were centrifuged and resuspended in full medium at the same temperatures. ZC3H11 was immunoprecipitated and the labelled protein detected by autoradiography. Quantitation was by scanning the autoradiogram. **C.** Half-life of in situ-tagged V5-ZC3H11 protein. Procyclic cells expressing V5-ZC3H11 from a modified endogenous locus were incubated either at 37°C or 41°C for 1 hour and processed as for (A). Detection was with anti-V5 antibodies and to aldolase as loading control. The V5 tag adds about 1.5 kDa to the protein molecular weight but seems to have a greater effect on migration of ZC3H11 in polyacrylamide gels. **D.** Half-life of ZC3H11-myc protein. Procyclic cells expressing ZC3H11-myc from a modified endogenous locus (native 3'-UTR was changed to actin 3’-UTR as a result of C-terminal in situ tagging) were grown at 27°C, and incubated either at 37°C or 41°C, or with 10μM sodium arsenite or 10μg/ml MG132 (MG) for 1 hour and processed as for (A). Detection was with anti-myc antibodies and Ponceau S staining is shown as loading control. The myc tag adds about 3 kDa to the protein molecular weight. **E, F.** Results from (A). Results are the mean ± standard deviation for three biological replicates were plotted and exponential curves were fitted to the mean values in Kaleidograph. The upper ZC3H11 band is designated "U" and the lower one "L". Ars = arsenite. **G.** Results from (B); for details see E,F. **H.** Results from (C); for details see E,F. **I, J.** Results from (D); for details see E,F. MG = MG132.

There was no change in the abundance of *ZC3H11* mRNA after heat shock [20]. To elevate the protein level, two options remained: increased mRNA translation, and increased protein stability. To address the latter possibility, we measured the half-life of ZC3H11. Since detection of the protein in unstressed cells was very unreliable, we compared cells that were incubated at either 37°C or 41°C, or with arsenite at 27°C. To measure the half-life we added cycloheximide, and followed the disappearance of the protein at the same temperatures by Western blotting. We quantified both detected species of ZC3H11 (Fig 2A, 2E and 2F). A minor cross-reacting band at about 50kD was very occasionally seen in unstressed control and *ZC3H11* RNAi samples (S1B Fig, lines 5–8) but was more commonly absent (Fig 2A, lane 15). At 37°C, the faster-migrating ZC3H11 protein (upper band) was slightly more stable than the slower-migrating protein (lower band) (Fig 2A, lanes 1–7, and 2E); at 41°C the lower band predominated and both bands were more stable than at 37°C (Fig 2A, lanes 8–14, and 2F; Table 1). Arsenite also strongly stabilized ZC3H11 (Fig 2E); only traces of the lower band were detected (Fig 2A, lanes 16–22; Table 1).

**Table 1:** Half-life measurements for ZC3H11. Results shown in Figs 2 and 3, and the replicate experiments, were analysed. For each individual experiment, the half-life of (tagged or untagged) ZC3H11 was measured. The mean and standard deviation for the 3 replicate experiments were then calculated. Because the calculation method was not the same, the results sometimes differ slightly from those shown in Fig 2E–2J.

	27°C	37°C-U	37°C-L	41°C-U	41°C-L	Ars	MG
35S-ZC3H11	nd	19±2	26±9	28±2	47±5		
ZC3H11	nd	9±4	14±3	^a^34±8, ^b^32±8	^a^138±52, ^b^126±25	93±21	
V5-ZC3H11	nd	14±4	nd	nd	178±31	134±23	
ZC3H11-myc	9±1	21±2	nd	nd	18±3	52±16	138±37
All	(9±1)	16±5	20±8	31±3	123±55*	93±41	
ZC3H11 CK RNAi				^b^84±22	^b^128±19		

^a^ Fig 2A

^b^ Fig 3D.

"nd" means that insufficient protein was detected for half-life measurement. A blank cell menas that the experiment was not done. "All" is the average of the result in the lines above

*excluding the result for ZC3H11-myc.

To rule out the possibility that the effects of temperature on stability were due to cycloheximide treatment, we measured degradation of ZC3H11 at 37°C and 41°C by pulse labelling with [^35^S]-methionine followed by a chase (Fig 2B). The half-life estimate for cells at 37°C was similar than that seen using cycloheximide (Table 1), with mostly the upper band present but some smearing (Fig 2B, lanes 2–7), whereas for 41°C there was a smear between roughly equal amounts of the upper and lower bands (Fig 2B, lanes 8–13), and the half-life estimate was shorter than after cycloheximide (Fig 2G and Table 1). The major difference between the two assays is that pulse-labelling examines only protein that was made in the previous 20 minutes, whereas the cycloheximide assay examines the complete pool of old and new protein. It is possible that the cycloheximide experiment detects a stable pool of ZC3H11 that has aggregated at 41°C, while the new soluble protein that is detected by pulse labelling is more accessible to degradation. The amount of ZC3H11 detected at 27°C was insufficient for half-life measurement by pulse labelling (Fig 2B, lane 1).

We also examined cells in which one *ZC3H11* locus had been tagged at the 5' end with sequence encoding a V5 tag. V5-ZC3H11 migrated exclusively as the upper band after incubation at 37°C (Fig 2C, lanes 1–7) and gradually shifted to the more prominent lower band after incubation at 41°C (Fig 2C, lanes 8–14). Half-lives were similar to those measured for the untagged protein (Fig 2H and Table 1). Arsenite again resulted in stabilization (Fig 2C, lanes 15–21, and 2H; Table 1).

To control for tag effects, a sequence encoding a myc tag, and a truncated actin (ACT) 3'-UTR, was inserted, by homologous recombination, at the 3'-end of the *ZC3H11* open reading frame. This results in an mRNA which encodes ZC3H11 with a C-terminal myc tag, and has an *ACT* 3'-UTR instead of the normal one. Interestingly, C-terminally myc-tagged ZC3H11 was detectable at 27°C (Fig 2D, lanes 1), but had lower abundance after incubation at 41°C (Fig 2D, upper panel lane 15) than at 37°C (Fig 2D, upper panel lane 7). After cycloheximide treatment, ZC3H11-myc had a half-life of about 10 min at 27°C, and was more stable at 37°C (Fig 2I). In contrast to previous results, a further temperature increase to 41°C had no effect on the apparent half-life; but the signal may have been too low for accurate measurement (Fig 2D). Arsenite (Fig 2D, lower panel lanes 7–12) and MG132 (Fig 2D, lower panel lanes 13–19) stabilized ZC3H11-myc (Fig 2J, Table 1)

Taken together, these results indicated that the increase in ZC3H11 abundance after heat shock treatment was at least partially due to an increase in protein stability. This effect was seen for both upper and lower bands, but the lower one was more stable than the upper one at all tested temperatures. Arsenite stabilised the slower-migrating (phosphorylated) species of ZC3H11. Since degradation was inhibited by MG132, the most likely effector is the proteasome. The results from the myc tagging also suggested that the *ZC3H11* 3'-UTR might play a role in temperature-dependent repression of ZC3H11 synthesis.

### Depletion of casein kinase I isoform 2 reduces ZC3H11 phosphorylation

We next wondered whether phosphorylation might play a role in ZC3H11 regulation. Although phosphorylation had previously been demonstrated unambiguously, we were unable to detect any phosphorylated peptides by mass spectrometry. It is possible that they are so negatively charged that they do not enter the mass analyser. This failure meant that we could not do a mutational analysis. As an alternative, we therefore looked for kinases and phosphatases that co-purified with tandem affinity purified ZC3H11 or MKT1 [21]. These were mitogen-activated protein kinase 2 (Tb927.10.5140), which co-purified with ZC3H11; and a protein phosphatase (Tb927.5.1660), an unclassified protein kinase (Tb927.5.2820) and casein kinase 1 isoform 2 (CK1.2, Tb927.5.800), which co-purified with MKT1. RNAi targeting the first two kinases and the phosphatase had no effect on ZC3H11 expression (S2B Fig). As previously demonstrated [25], targeting Tb927.5.2820 or Tb927.5.1660 did not affect trypanosome proliferation or morphology, whereas Tb927.10.5140 was essential.

**Fig 3:**
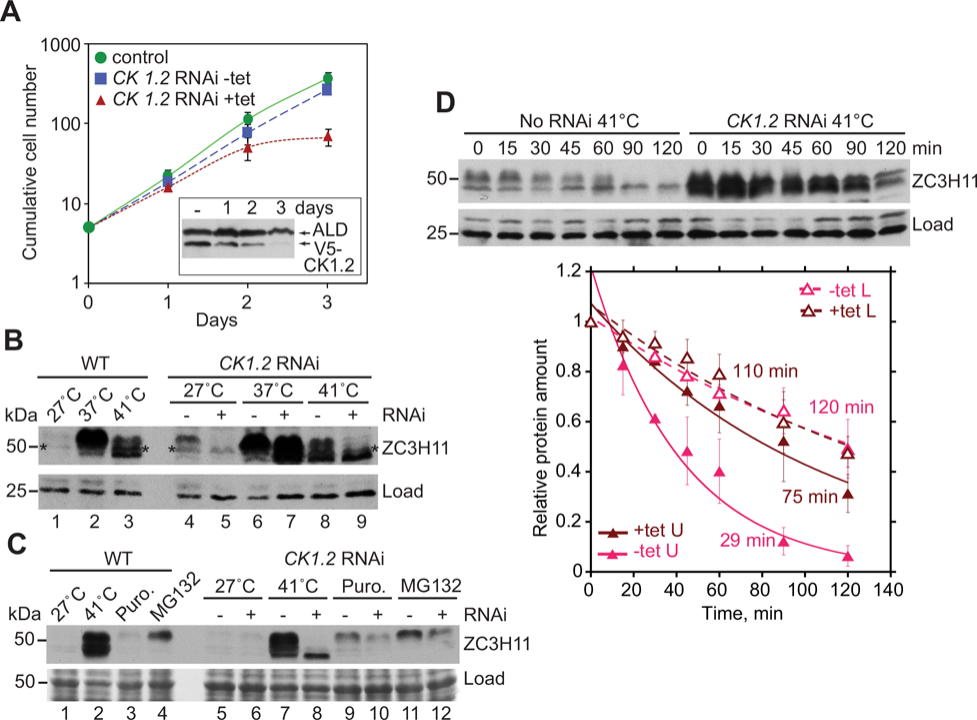
Casein kinase 1.2 depletion decreases ZC3H11 phosphorylation. **A.** Effect of casein kinase 1 isoform 2 (CK1.2) RNAi on proliferation of procyclic trypanosomes. Procyclic cells expressing in situ tagged V5-CK1.2, with a tetracycline-inducible stem-loop RNAi construct, were induced with tetracycline (200g/ml). The cumulative cell counts (numbers of cells that arose from every 5 cells, accounting for dilution) are shown as arithmetic mean ± standard deviation of three experiments. The parental cell line without tetracycline was used as a control. The inset shows the level of V5-CK1.2 in cells with and without tetracycline, analysed by Western blotting. 5×10^6^ cells were loaded per lane. Aldolase (ALD) served as loading control. **B.** CK1.2 depletion causes a decrease in phosphorylated ZC3H11. RNAi was induced for 2 days, then the cells were transferred to either 37°C or 41°C for 1 hour. ZC3H11 was detected by Western blotting, with a 25kD cross-reacting protein that is unaffected by heat shock as loading control. Cytoskeleton-free extracts from 5×10^6^ cells were loaded per lane. *could be a cross-reacting protein or a species with intermediate phosphorylation. One representative image from three separate experiments is shown. **C.** Dephosphorylated ZC3H11 does not accumulate in CK1.2 depleted cells treated with puromycin or MG132. The experiment is as in (B), except that the Ponceau S staining serves as the loading control. **D.** Effect of CK1.2 depletion on ZC3H11 turnover after severe heat shock. Cells with or without 2 days of RNAi induction were subjected to a 1 hour heat shock at 41°C, then cycloheximide (100μg/ml) was added. Details are as in Fig 2. Quantification of the two bands after RNAi was difficult since they were not well separated.

Depletion of CK1.2 inhibited cell growth (Fig 3A). The effect was beginning to be visible after 2 days, when the level had only decreased to about 40% of normal (Fig 3A, inset). CK1.2 is also essential in bloodstream form *T*.*brucei* [26]. CK1.2 depletion caused a decrease in the relative abundance of the slower-migrating (phosphorylated) ZC3H11 species, irrespective of the temperature (Fig 3B, lanes 5, 7 and 9) or the stress applied (Fig 3C, lanes 8, 10 and 12). After heat shock, dephosphorylated ZC3H11 persisted in the CK1.2-depleted cells (Fig 3B, lanes 7 and 9, and 3C, lane 8); it was not seen after the other stresses (Fig 3C, lanes 10 and 12). After a 41°C heat shock, the half-life of the dephosphorylated ZC3H11 was the same with or without *CK1*.*2* RNAi (Fig 3D). We concluded that phosphorylation may play a role in destabilizing ZC3H11. The upper band also seemed to have become more stable, although separating them for measurement was rather difficult.

**Fig 4:**
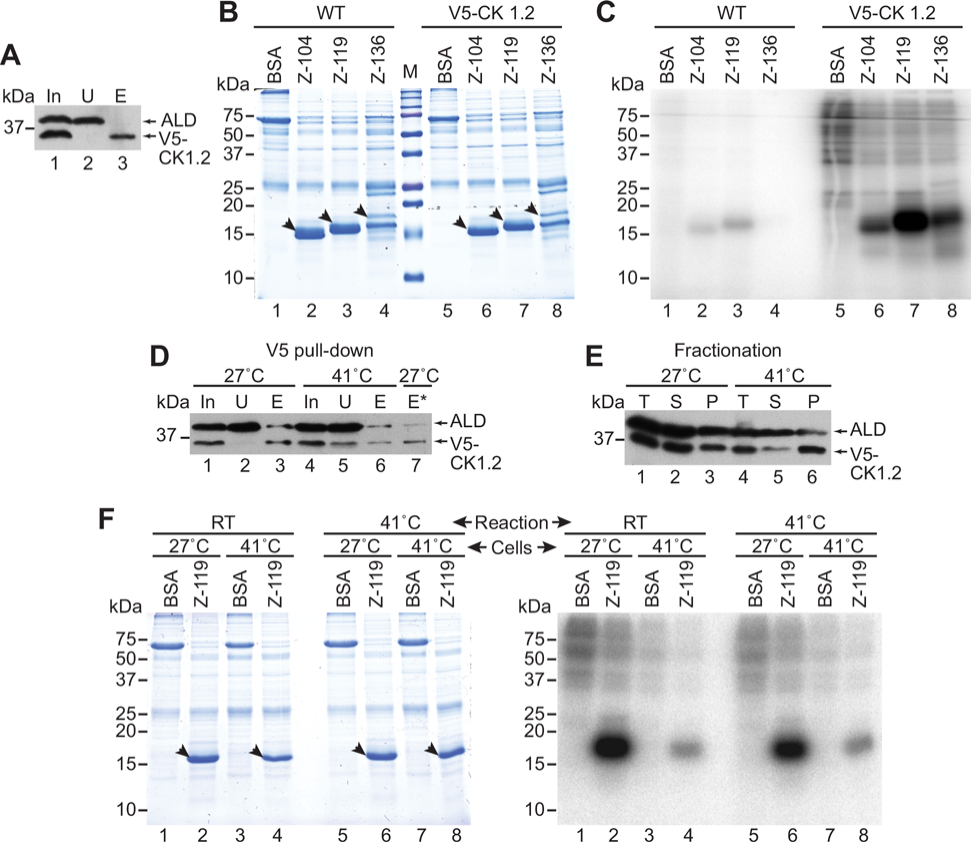
V5-tagged CK1.2 can phosphorylate ZC3H11. **A.** An extract from 1.5×10^7^ procyclic cells expressing V5-CK1.2 was subjected to immunoprecipitation with anti-V5 beads. The efficiency of pull-down was analysed by Western blotting using anti-V5 and anti-aldolase (ALD) as a control. In: input, U: unbound (5×10^6^ cell-equivalents), E: eluate (5×10^6^ cell-equivalents). The beads were used for the in vitro kinase assay. **B.** Recombinant His_10_-ZC3H11 N-terminal fragments (first 104, 119 or 136 amino acids) were purified from E. coli, then 5μg of the preparation were added to the V5-CK1.2 beads from (A) (lanes 2–4 and 6–8). 5μg of commercial BSA (lanes 1 and 5) was used as a negative control. After incubation with [γ-^32^P]-ATP, the supernatants were subjected to SDS-PAGE. The arrows show the full-length proteins on the Coomassie-stained gel. "WT" (lanes 1–4) is the negative control: anti-V5 beads incubated with trypanosome lysates that did not contain any V5-tagged protein. Lanes 5–8 were incubated with V5-CK1.2. **C.** Autoradiogram of the SDS-PAGE analysis in (B). A second experiment gave the same result. **D.** Purification of V5-CK1.2 from cells grown at 27°C or after a one-hour heat shock. For details see (A).To compensate the lower efficiency of pull-down from heat-shocked cells two times less beads from control cells were used for the kinase assay (E*, lane 7). **E.** Fractionation of V5-CK1.2-expressing cells, with or without a one-hour heat shock, in order to assess distribution of V5-CK.1.2 in supernatant and insoluble cell debris. **F.** Kinase assay as in (A-C), with the119-residue ZC3H11 fragment as substrate (lanes 2, 4, 6, 8), and V5-CK1.2 from normal (lanes 1–2, 5–6) or heat-shocked cells (lanes 3–4, 7–8) as the enzyme. BSA (lanes 1, 3, 5, 7) was the negative control. Reactions were incubated either at room temperature (RT, lanes 1–4) or at 41°C (lanes 5–8) for 20min.

We could not mutate the *in vivo* phosphorylation sites of ZC3H11, since they are unknown. We therefore could not directly assess the contribution of phosphorylation to ZC3H11 instability. Equally, we could not tell whether the effects of CK1.2 depletion were direct or indirect. We could, however, at least test whether ZC3H11 could serve as a substrate for CK1.2. For this, recombinant N-terminal fragments of Z3H11 (first 104, 119 or 136a.a.) purified from *E*.*coli* via a His_10_-tag, were incubated with immunoprecipitated V5-CK1.2 (Fig 4A) in the presence of [γ-^32^P]-ATP. The products were resolved by SDS-PAGE (Fig 4B) and visualized by autoradiography (Fig 4C). All three ZC3H11 fragments were phosphorylated by CK1.2, but to different extents (Fig 4C, lanes 6–8). The 119a.a. fragment gave the strongest signal (Fig 4C, lane 7) but the amount of the purified 136a.a. fragment loaded was lower, because it was only partially soluble and the preparation contained contaminants (Fig 4B, lanes 4 and 8). These results show that CK1.2 could be directly responsible for ZC3H11 phosphorylation, but an indirect role is equally possible. We considered testing the effect of CK1.2 RNAi on the heat shock response, but concluded that the results would not be meaningful because CK1.2 RNAi compromises cell viability.

### Casein kinase I isoform 2 is inactivated at 41°C

We next asked why ZC3H11 phosphorylation was decreased by severe heat shock. To find out whether CK1.2 was inactivated, we wanted to compare the *in vitro* activities of V5-CK1.2 obtained from cells at 27°C or after one hour at 41°C. When we did the V5-CK1.2 pull-downs from extracts from cells treated at 41°C, it appeared that only some of the V5-tagged enzyme was accessible to the anti-V5 antibodies (Fig 4D, compare lanes 5 & 6 with lanes 2& 3). To investigate the reason for this, we analyzed the soluble supernatant fraction (input for immunoprecipitation) and cell debris fraction (pellet). CK1.2 was normally partially in the pellet (Fig 4E, compare lanes 2 and 3), but after incubation at 41°C, the proportion in the pellet reproducibly increased (Fig 4E, compare lanes 5 and 6 with lanes 2 and 3). The lowered pull-down efficiency could therefore be due to masking of the V5 tag within aggregates. To compensate for this for the kinase assay, we therefore took amounts of purified material that were predicted to contain roughly the same amounts of enzyme rather than the same cell equivalents (Fig 4D, lane 7). (Note that since the assay uses freshly prepared protein we could not test the CK1.2 content in advance.) Phosphorylation of the 119-residue ZC3H11 fragment was substantially reduced when the V5-CK1.2 purified from heat-shocked cells was used (Fig 4F, compare lanes 2 and 4). In fact the residual activity was not much greater than the background that we had previously seen from cells that did not express V5-CK1.2 (Fig 4C, lane 3). The result was not much affected if the kinase assay was performed at 41°C (Fig 4F, compare lanes 2 and 6), suggesting that some of the loss in CK1.2 activity depended on the cellular environment.

These results suggested that the decrease in ZC3H11 phosphorylation upon heat shock could be due to inactivation of CK1.2.

### Stress promotes translation of the ZC3H11 mRNA

**Fig 5:**
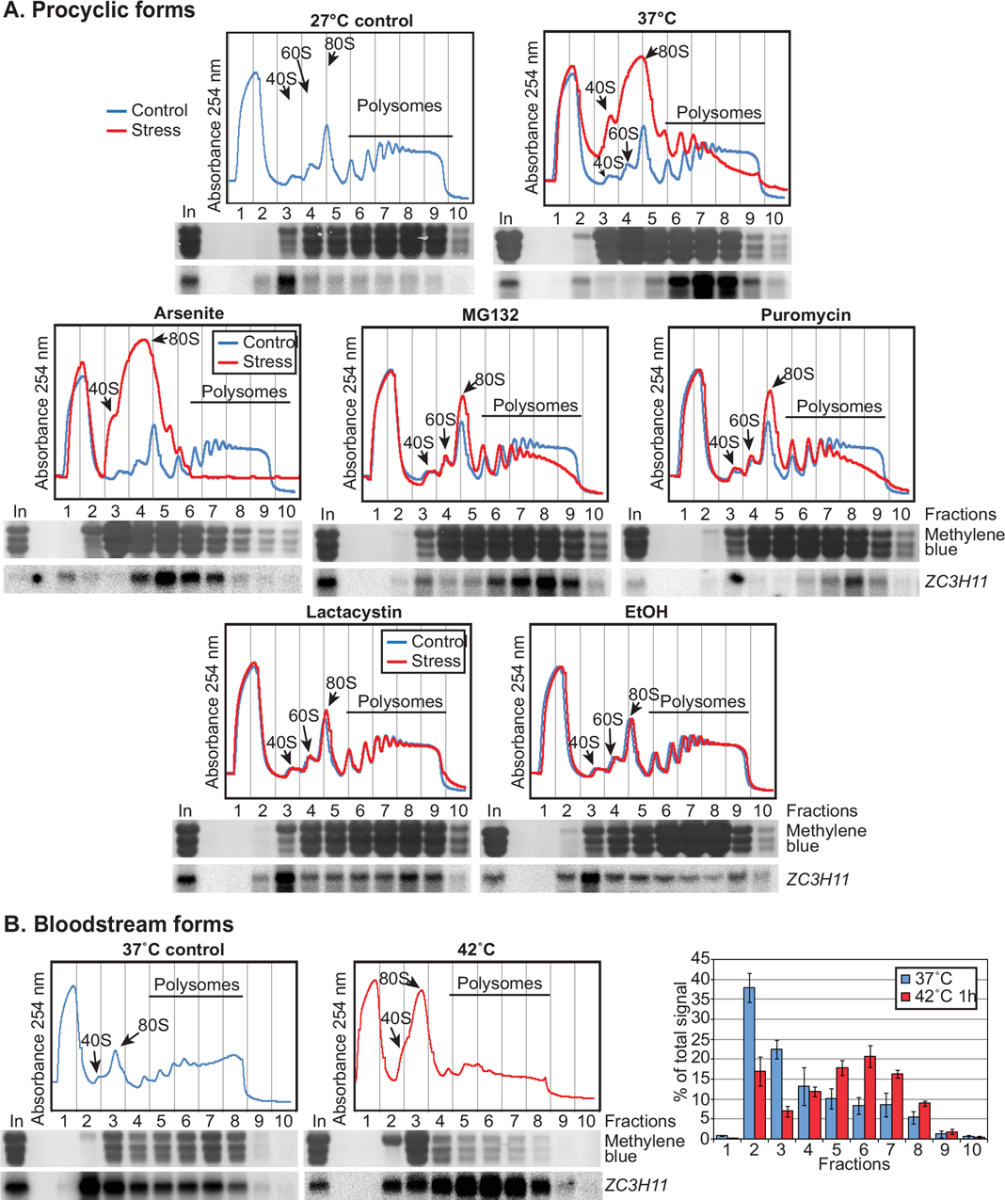
Stress promotes translation of ZC3H11 mRNA. **A.** Procyclic cultures were grown under normal conditions and after different treatments, then extracts were subjected to sucrose gradient centrifugation. The upper panels show representative absorbance profiles at 254 nm, and the lower panels are Northern blots of RNA preparations from the different fractions. The stresses were all for 1 hour: 37°Cheat shock, puromycin (1μg/ml), proteasome inhibitors MG132 (10μg/ml) and lactacystin (10μM), arsenite (10μM) and ethanol (2%). Peaks for small (40S), large (60S) ribosomal subunits, monosomes (80S) and polysomes are indicated with arrows. A methylene blue stain for a typical profile is shown beneath the fraction numbers. *ZC3H11* mRNA was detected in all of the blots. Results from one representative experiment out of several repeats are shown. Examples of quantifications for 27°C and 37°C are in Fig 6. **B.** Effect of heat shock on *ZC3H11* mRNA translation in bloodstream forms. Everything is as in (A) except that the temperatures are different. The lower panel shows the quantification. The mRNA signal from each fraction was quantified by phosphorimaging, and expressed as a percentage of the total signal. Results are arithmetic mean ± standard deviation for three biological replicates.

Although heat shock clearly affected ZC3H11 stability, the 2–3 fold change in half-life seemed unlikely to be sufficient for the major differences in steady-state protein that were observed. Moreover, we knew that replacing the 3'-UTR of the *ZC3H11* resulted in increased protein abundance at 27°C, and diminished abundance at 41°C (Fig 2D). We therefore turned our attention to ZC3H11 protein synthesis: we examined the association of the *ZC3H11* mRNA with polysomes by sucrose gradient centrifugation. Startlingly, at 27°C the *ZC3H11* mRNA was concentrated in the low-density region of the gradient, co-migrating with the 40S small ribosomal subunits (Fig 5A, 27°C control, fraction 3). After a mild heat shock of 37°C for 1 hour, as expected, the overall distribution of ribosomes had shifted, with an increase in 80S at the expense of polysomes (Fig 5A, 37°C). The same shift was also seen at 41°C (S3A Fig), and with puromycin, arsenite and MG132, but not with lactacystin or ethanol (Fig 5A). After 37°C heat shock, puromycin, arsenite or MG132 treatment the *ZC3H11* mRNA had shifted almost completely to the polysomal fractions (Fig 5A, fractions 6–9). This suggests that the increase in ZC3H11 protein after heat shock, puromycin, arsenite and MG132 treatment is partly caused by increased protein synthesis. In contrast, the increases in ZC3H11 after lactacystin and ethanol are probably mainly due to protein stabilization, since these two treatments had only very minor effects on *ZC3H11* translation (Fig 5A).

Inclusion of translation inhibitors can cause accumulation of 80S ribosomes near the start codon [27]. Although we cannot really imagine how this could cause association of *ZC3H11* mRNA with a 40S fraction, we nevertheless felt that it was essential to make sure that the same behaviour was seen without cycloheximide. As expected, without cycloheximide, the 80S peak was higher and polysomes were slightly decreased (S3B Fig). *ZC3H11* mRNA, however, stayed in the 40S fraction.

The silenced *ZC3H11* mRNA appeared to be migrating with the 40S ribosomal subunit. We decided to examine this behaviour in more detail. We used a 10%-30% sucrose gradient, which gives better resolution of ribosomal subunits and monosomes than a 17.5%-50% gradient, and took more fractions (S3C Fig). *ZC3H11* mRNA still migrated at 40S. One possible explanation is that the *ZC3H11* mRNA might be associated with 40S ribosomal subunits, which would either be cap-associated, scanning the 5'-UTR or "stuck" at the start codon. This seemed somewhat unlikely since initiation complexes usually migrate at 48S. Alternatively migration at this position could be due to many proteins binding along the mRNA. We attempted to distinguish these possibilities by digesting the *ZC3H11* RNA with RNase H prior to gradient analysis (S3C Fig). We cut the RNA into three pieces containing the 5' end, to look for 40S association with the cap; a region around the start codon, to look for a block in 60S joining; and the remaining 2.4 kb which should not be associated with a small subunit. If just one mRNA contained a bound 40S subunit, it should migrate lower in the gradient than the others. Instead, each piece migrated at a density that was less than 40S, but higher than the main protein peak. This result suggests that the migration of the intact mRNA at 40S was not due to specific association of a charged 40S subunit with the cap, 5' UTR, or start codon. However, we cannot rule out the possibility that bound 40S subunits dissociated during the RNase H digestion.

There is some evidence that expression of the *HSP83* mRNA in *Leishmania mexicana* is regulated by changes in the secondary structure of a sequence in the 3'-UTR [16]. The fact that *ZC3H11* translational regulation was seen in procyclic trypanosomes after a variety of stresses at 27°C, however, argued against any role for changes in mRNA secondary structure. Most compellingly, in bloodstream forms growing at 37°C, *ZC3H11* mRNA was largely in the 40S fraction (Fig 5B, left panel). Meanwhile in bloodstream forms at 42°C, when most translation had been suppressed, *ZC3H11* mRNA was in the polysome fraction (Fig 5B, right-hand panel). This unambiguously demonstrated that the increase in *ZC3H11* translation was a response to *abnormally increased* temperature, rather than to the temperature *per se*.

### ZC3H11 UTRs control translation

**Fig 6:**
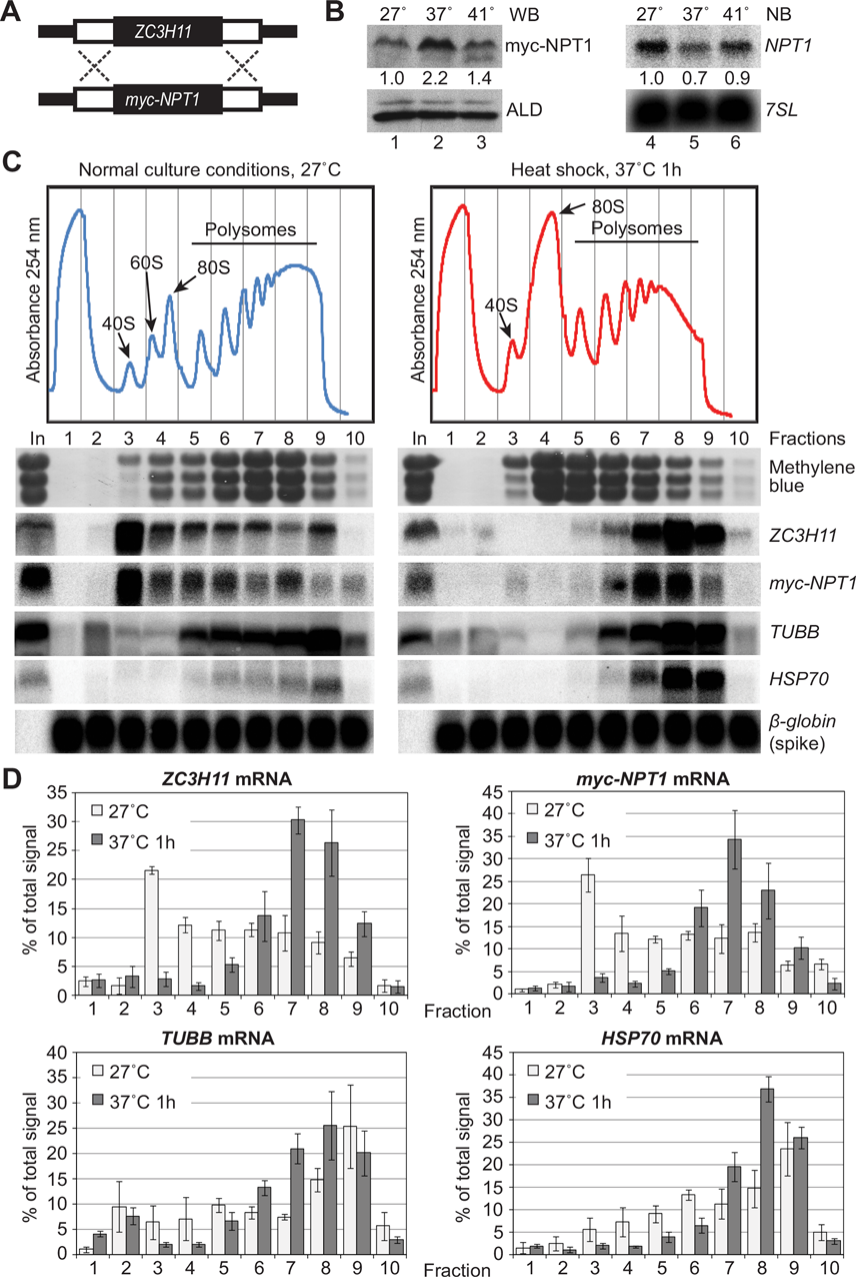
The *ZC3H11* UTRs regulate translation upon heat shock. **A.** Schematic representation of the replacement of a ZC3H11 ORF by the neomycin resistance marker (myc-NPT1) in procyclic forms. **B.** Western and Northern blot analysis of myc-NPT1 gene expression at 27°C and after mild (1h at 37°C) or severe (1h at 41°C) heat shock. One representative example is shown. Aldolase (ALD) was used as a loading control for western blotting and 7SL (signal recognition particle RNA) for Northern blotting. **C.** Representative polysome profiles of cells expressing myc-NPT, with or without heat shock. Details are as for Fig 5A. In vitro transcribed human β-globin RNA was added as a spike-in to each fraction before RNA preparation to check preparation efficiency. **D.** Quantification of results for 3 biological replicates, as described in Fig 5B.

To find out whether the *ZC3H11* coding region plays any role in translation control, we replaced one *ZC3H11* open reading frame with a gene encoding neomycin phosphotransferase (NPT1) with an N-terminal myc tag (Fig 6A). (This leaves the other allele intact.) Both UTRs were preserved after this knock-in procedure. After one hour of mild heat shock at 37°C (Fig 6B, lane 2), the myc-NPT1 protein level was increased 2-fold in comparison to the control, while mRNA levels were slightly decreased (Fig 6B, lane 5). Polysome profiling revealed that *myc-NPT1* mRNA completely mimicked the behaviour of *ZC3H11* mRNA (Fig 6C). The *ZC3H11* open reading frame was therefore not required for translational regulation. Quantification (Fig 6D) revealed that at 27°C, less than 35% of the *ZC3H11* or myc-*NPT1* mRNA was in the heavy polysomes (fractions 5–10) while more than 20% migrated at 40S (fraction 3). After a 37°C heat shock, 60–65% of both *ZC3H11* and *myc-NPT1* mRNAs was in heavy polysomes (Fig 6D). The distribution of β-tubulin mRNA (*TUBB*) along the gradient did not change in response to heat shock, but *HSP70* mRNA had accumulated in heavy polysome fractions.

### A region of 71 nucleotides in the ZC3H11 3'-UTR represses translation

**Fig 7:**
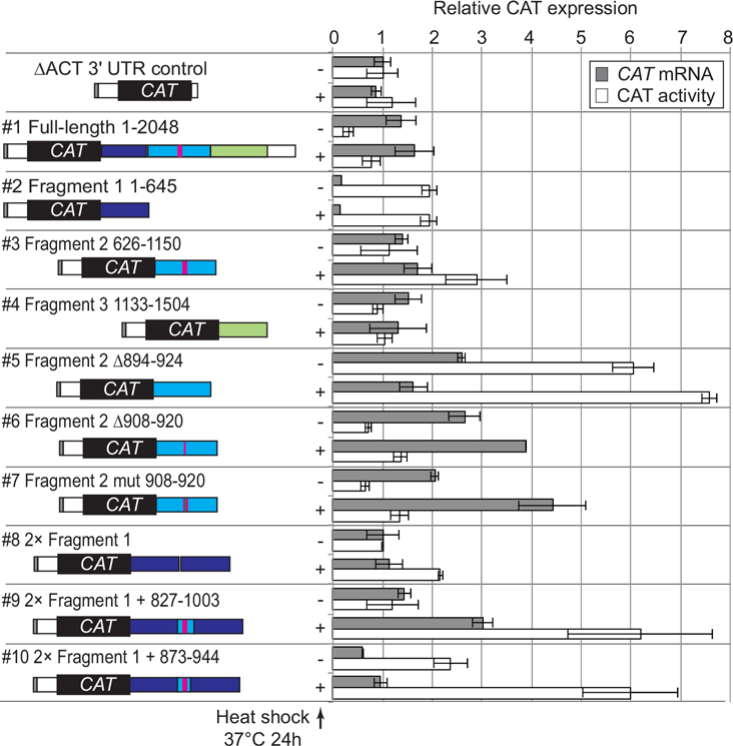
A 71nt portion of the *ZC3H11* 3'-UTR is required for heat-regulated suppression of expression. The left panels are diagram of CAT reporter constructs for *ZC3H11* 3'-UTR analysis (shown not to scale). The CAT ORF is flanked by the EP 5'-UTR and full-length or different fragments of the *ZC3H11* 3'-UTR. Procyclic cell lines constitutively expressing CAT reporters, integrated into the tubulin locus, were generated. Numbers above the diagrams indicate nucleotide positions on the full-length *ZC3H11* 3'-UTR. The graphs show CAT mRNA and CAT activity levels, with or without 37°C 24h heat shock as arithmetic mean ± standard deviation of 3 independent measurements. A truncated actin 3'-UTR (∆ACT) was used as the control for constructs #1–7. For #9 and #10, the control was the parent construct #8 with 2 copies of fragment 1 separated by multi-cloning site.

**Fig 8:**
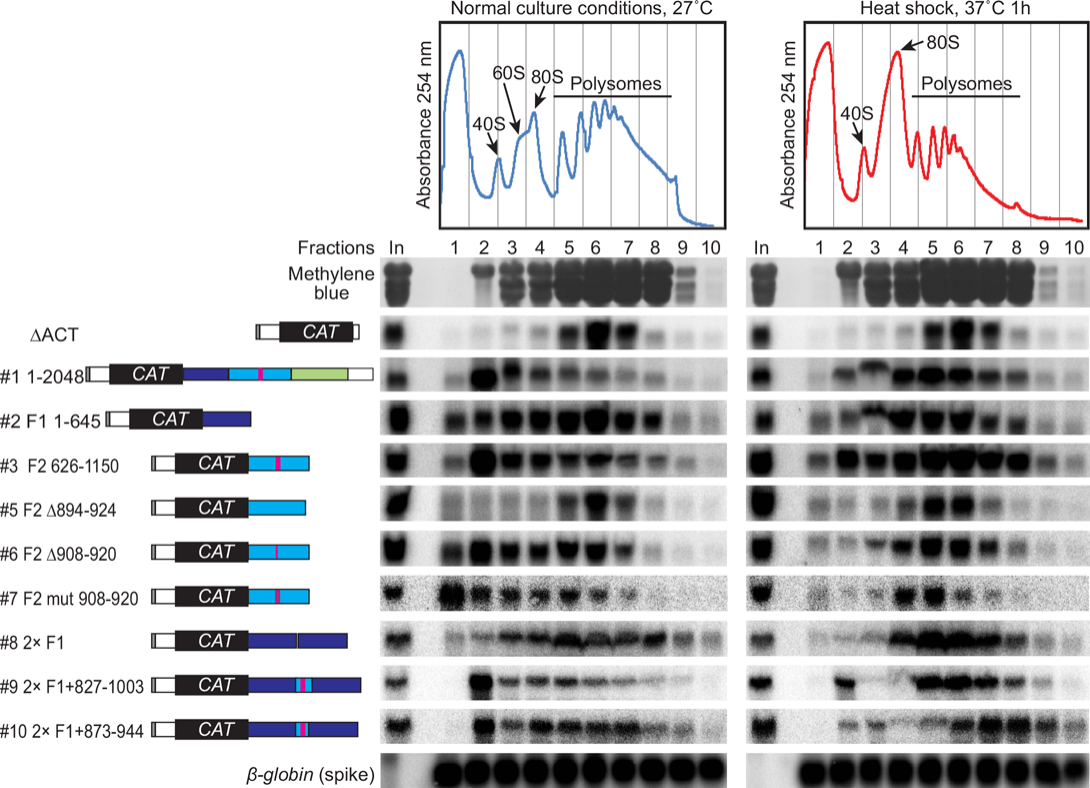
A 71nt portion of the *ZC3H11* 3'-UTR is required for heat-regulated translation repression. The top panels show representative 254nm absorbance profiles from sucrose density gradients using extracts from procyclic trypanosomes grown at 27°C, or shifted for one hour to 37°C. Peaks for small (40S), large (60S) ribosomal subunit, monosome (80S) and polysomes are indicated with arrows and the rRNA staining with methylene blue is shown below. The lines show the times at which the fractions shifted, which do not correspond exactly to the tube contents. The panels below show Northern blot detection of CAT mRNA. In vitro transcribed human β-globin RNA was added as a spike-in control for equal RNA isolation efficiency. Results from one representative experiment out of three separate repeats are shown.

To find out whether the *ZC3H11* 3'-UTR alone could confer a response to heat shock, we made a reporter plasmid in which the chloramphenicol acetyltransferase (*CAT*) ORF was flanked by the *EP* 5'-UTR (from a gene encoding EP procyclin) and the *ZC3H11* 3'-UTR (Fig 7, left-hand panel). The construct was designed for integration into the tubulin locus, which will result in transcription by RNA polymerase II. Procyclic cells containing the reporter were either incubated at 27°C, or stressed at 37°C for 24 hours, then CAT protein activity and *CAT* mRNA levels were measured (Fig 7, right-hand panel). All results were normalized to those from a control CAT construct with a truncated actin 3'-UTR (∆ACT). In addition, translation efficiencies were estimated by polysome profiling (Fig 8). In comparison to the control reporter, the full-length *ZC3H11* 3'-UTR construct showed approximately 3-fold decrease in CAT activity at 27°C, which was more than doubled after heat stress (Fig 7, construct #1). In contrast, *CAT* mRNA levels were comparable with those from the ∆*ACT* control and did not change much at 37°C (Fig 7, construct #1). The control *CAT* reporter was in the polysomes at both temperatures (Fig 8, top panel) whereas the *CAT* reporter with the *ZC3H11* 3'-UTR was, like native *ZC3H11*, concentrated in the 40S fraction at 27°C and in the polysomes at 37°C (Fig 8, construct #1). The reporter was thus mimicking the behaviour of the native *ZC3H11* mRNA.

In order to localise sequences that were required for translation control, we now started to test different segments of the *ZC3H11*3'-UTR. Results for CAT activity and mRNA are shown in Figs 7 and S4A, and corresponding polysome gradients are in Figs 8, S4B and S4C. Fragment #1 (nt 1–645 relative to the stop codon, construct #2), and fragment #3 (nt 1133–1504, construct #4) were not able to give regulation, but a reporter with fragment #2 (nt 626–1150, construct #3) gave the same pattern as the full-length 3'-UTR. Several stem-loop structures were predicted within this segment (S5A Fig). Deletion of a predicted stem-loop at nt 894–924 (S5A Fig), shown as red bar in Fig 7, caused 6-fold increase of CAT activity and 2.6 fold increase of the CAT mRNA levels at 27°C (Fig 7, construct #5), eliminating the response to elevated temperature and causing a complete loss of translational repression at 27°C (Fig 8, construct #5). Deletions of two other predicted stem-loops (945–982 or 1003–1076) resulted in strong decrease in the reporter mRNA levels, but both mRNAs still showed translational activation at 37°C (S4B and S4C Fig). The 894–924 segment has a GU-rich sequence—GUUGUUGUUGUUG—at positions 908-920.Deletion of this sequence (construct #6) or mutation of the Gs to Cs (CUUCUUCUUCUUC, construct #7), were both predicted to eliminate the stem-loop as shown by Mfold (S5B Fig). Both of these mutations gave only a marginal attenuation of the translational block at 27°C (Figs 7 and 8).

Our results so far indicated that the sequence from 894–924 was necessary for temperature-dependent translational repression. We next investigated whether this sequence was also sufficient to give regulation. Insertion of the sequence into the control reporter, between the *CAT* ORF and the *ACT* 3'-UTR had no effect, and placing it between two copies of the *ACT* 3'-UTR (100nt each) again gave no regulation. However, incorporation of nts 827–1003 (construct #9) or 873–944 (construct #10) between 2 copies of fragment #1 led to translational repression similar to that from the full-length fragment #2. Thus the 71nt from positions 873–944 in the *ZC3H11* 3'-UTR were only sufficient for temperature-dependent translational regulation in a particular context. We do not know whether it is the sequence of fragment #1, or simply the distance from the poly(A) tail or termination codon, that is important for the function of the 71nt element.

### Translationally repressed ZC3H11 mRNA is not in large RNP granules

**Fig 9:**
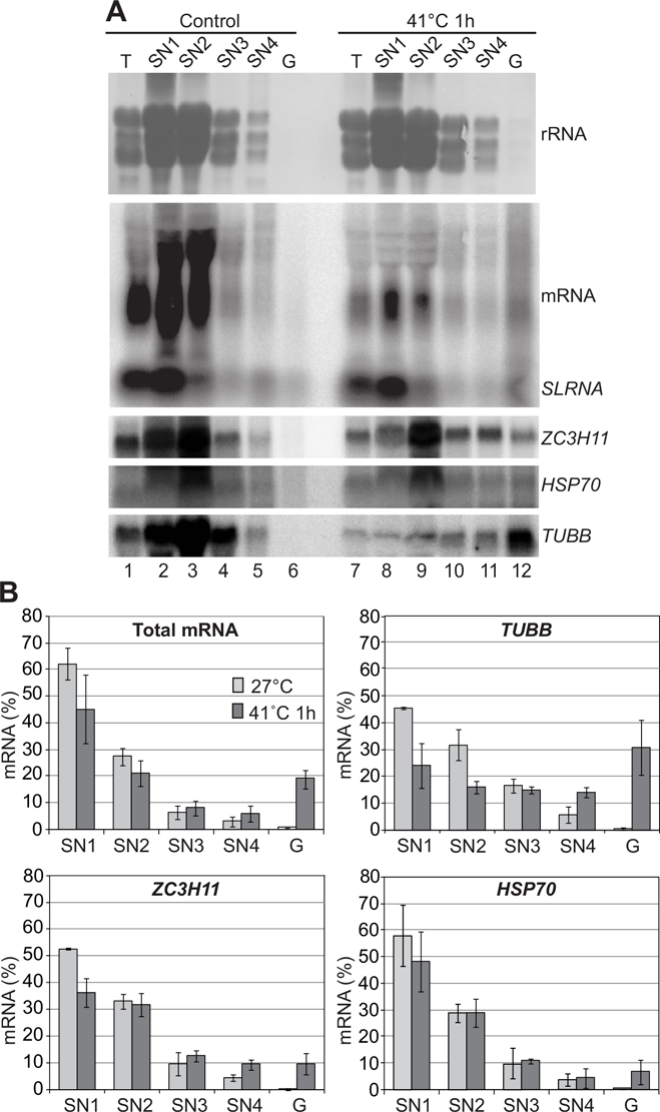
Incorporation of mRNAs into granules after severe heat shock. **A.** Analysis of RNA samples taken during the granules enrichment. Equal volume of the RNA samples (except SN1) isolated from untreated and heat-shocked procyclic cells were probed for total mRNA, *ZC3H11*, HSP70 and β-tubulin mRNAs. For SN1 samples only half of the volume was loaded to prevent gel overloading. ‘T’ is RNA sample prepared in parallel as a control for RNA quality and is not equivalent to the other samples. One representative experiment out of three separate repeats is shown. **B.** The percentage of the total mRNA, *ZC3H11*, HSP70 and β-tubulin mRNAs (mean ± standard deviation for three independent experiments) quantified from Northern blots (A). This percentage is calculated based on total mRNA signal being the sum of SN1, SN2, SN3, SN4 and G.

Association of *ZC3H11* mRNA in small cytosolic granules [14] might conceivably result in migration in the 40S-80S range. To test this we employed the novel method for granule enrichment described in [28]. This method exploits the trypanosome sub-pellicular microtubule corset as a natural sieve to trap structures larger than about 24 nm. After cell lysis under conditions that maintain the microtubules, all macromolecules in lower-diameter structures are released during 3 wash steps (Fig 9A, SN1-3); these washes contain most of the ribosomal RNA and 60–80% of total mRNA (Fig 9A and 9B). The microtubules are then disrupted by high salt; and the released material is separated into a small-granule fraction (S4) and a large granule pellet fraction (G in Fig 9).

At 27°C, only about 5% of the β-tubulin (*TUBB*) mRNA was in the small and large granule fractions, but after a 41°C heat shock, the amount in small granules had doubled and 30% was in the large granule fraction (Fig 9). This result suggests that the procedure is able to enrich heat shock granules. The accumulation of *TUBB* mRNA in granules was specific: only 10% of *HSP70* mRNA was in granules after heat shock (Fig 9), which is expected since the *HSP70* mRNA retains active translation. The fraction of total mRNA in granules after heat shock was lower than for tubulin, which might be because after heat shock, the actively translated *HSP* mRNAs are preferentially stabilized whereas other mRNAs are lost. There was no evidence that *ZC3H11* mRNA is in granules larger than 24nm at 27°C: less than 5% was in the small granules and none was in large granules (Fig 9). After heat shock almost 10% of the *ZC3H11* mRNA was enriched in small and large granule fractions—slightly more than for *HSP70*, but considerably less than for *TUBB*.

**Fig 10:**
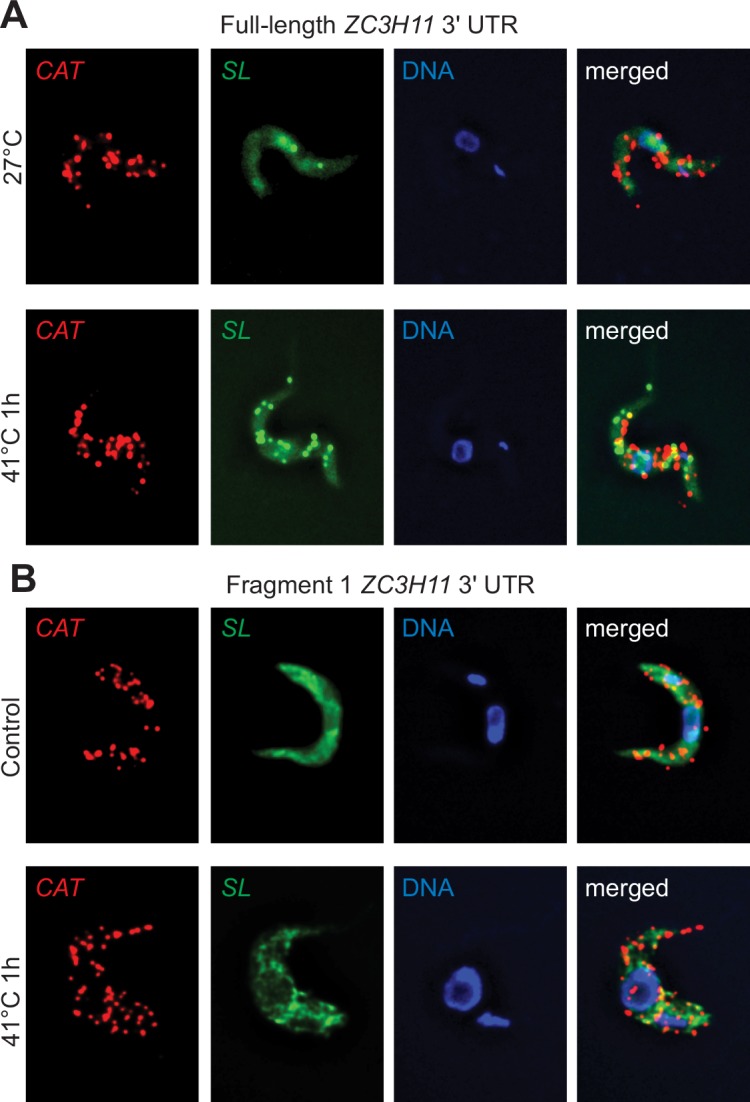
mRNAs with *ZC3H11* 3'-UTRs are distributed throughout the cytosol. Single mRNA FISH was done using Affymetrix probes. Procyclic cells expressing the CAT reporter with full-length *ZC3H11* 3'-UTR (A) or fragment 1 of the *ZC3H11* 3'-UTR (B) were heat-shocked for 1 hour at 41°C, or left at 27°C. Slides were prepared and hybridized with a probe sets antisense to the CAT ORF (red) and the spliced leader (SL) probe (green) together with DAPI staining (blue). One representative image of untreated and heat-shocked cells out of several experiments is shown.

We also checked whether the *ZC3H11* 3'-UTR can influence the subcellular location of a reporter mRNA by single-molecule *in situ* hybridisation. The locations of *CAT* mRNAs with either the full *ZC3H11* 3'-UTR, or the proximal 645nt fragment #1, which does not give regulation, were compared with that of total mRNA (detected with an *SL* probe). At 27°C the *SL* probe gave a very even cytosolic signal and both *CAT* mRNAs were scattered throughout the cytosol (Fig 10). After heat shock, some concentration of the total mRNA was clearly evident but the localisations of the two *CAT-ZC3H11* mRNAs were unchanged (Fig 10). The results must be treated with some reservation because it is possible that *CAT* mRNAs in granules were not very accessible to the rather large probes. Similarly, if there are hundreds of very small suppressive granules, we would not have seen clustering of the *CAT* mRNAs, since there were less than 50 *CAT* molecules per cell. Despite these caveats, our results yielded no evidence for a role for granules in regulation by the *ZC3H11* 3'-UTR.

## Discussion

The amount of ZC3H11 was increased by stresses that cause the accumulation either of protein fragments, or of incompletely folded proteins in the cytosol. ZC3H11 protein abundance was, in contrast, *not* affected by inducers of ER stress. This result corresponds to the role of ZC3H11 in stabilizing mRNAs that encode proteins involved in refolding cytosolic proteins [20]. The pattern of induction suggests that the existence of (partially) unfolded proteins might be the stimulus that causes ZC3H11 accumulation. However, there is a conundrum, since ZC3H11 expression is increased at 37°C in procyclic forms, but repressed at the same temperature in bloodstream forms. Putting procyclic forms at 37°C is most unlikely to cause mass protein denaturation, since the bulk of the procyclic proteome is identical to that of bloodstream forms. It is however possible that some procyclic-specific proteins are heat-sensitive.

How meaningful is the increase in ZC3H11 expression? The total number of mRNA target molecules per procyclic trypanosome is not greater than 500 [2,20], and at 27°C there were about 2×10^3^ ZC3H11 molecules per cell—a 4:1 ratio. However, the (AUU) repeats in the target mRNAs should be able to bind several ZC3H11 molecules. It is therefore possible that at 27°C, not all binding sites are occupied. In contrast, full occupancy would be mathematically possible after heat shock, when ZC3H11 levels have increased almost 10-fold. Gel-shift experiments with a recombinant ZC3H11 fragment gave a dissociation constant of about 30nM, which is similar to the concentration of ZC3H11 after heat shock. Thus the difference in concentration is indeed meaningful. It is also possible that phosphorylation of ZC3H11 affects RNA binding.

Under normal growth conditions ZC3H11 protein is rapidly degraded. Degradation is probably by the proteasome, since lactacystin increases the amount of ZC3H11 (Fig 1A, lane 7) without affecting *ZC3H11* mRNA translation (Fig 5A). MG132 inhibits ZC3H11 degradation (Fig 2D) as well as activating *ZC3H11* translation (Fig 5A). Both of these agents inhibit the proteasome, although lactacystin is thought to be more specific [29]. We do not know why only one of them induced *ZC3H11* translation, but it is possible that at the doses used, lactacystin gave either less complete proteasome inhibition, or had fewer side-effects than MG132. After appropriate stresses, degradation of ZC3H11 is inhibited. As noted above, it is unlikely that this is solely because the proteolytic system is overloaded with stress-insulted proteins, at least in procyclic forms at 37°C. A more specific effect such as a change in ZC3H11 modification seems more probable. After treatment with MG132 and puromycin, phosphorylated ZC3H11 accumulated (Fig 3). In contrast, severe heat shock caused a very strong increase in dephosphorylated ZC3H11, which was more stable than the phosphorylated version. The dephosphorylated version is likely to be functional, since we know that unmodified ZC3H11 can bind to RNA and can interact with MKT1, PBP1 and itself [21].

To analyse the role of ZC3H11 phosphorylation, we looked for candidate kinases. Of the three kinases that co-purified with affinity tagged ZC3H11, we found evidence for possible involvement of one: CK1.2. Two lines of evidence implicate CK1.2 in ZC3H11 phosphorylation. The first is that depletion of CK1.2 results in accumulation of faster-migrating ZC3H11, consistent with partial loss of phosphorylation (Fig 3). The second line of evidence is that CK1.2 can phosphorylate N-terminal fragments of ZC3H11 *in vitro* (Fig 4). We therefore speculate that the accumulation of dephosphorylated ZC3H11 after severe heat shock could be due to inactivation of CK1.2. Other kinases could however also be involved: depletion of CK1.2 is ultimately lethal, and could easily lead to the loss of other kinases. Bloodstream-form trypanosomes lacking the MAP kinase kinase homologue, MKK1, were impaired in the ability to grow at 39°C [30], and procyclic cells lacking the MAP kinase homologue MAPK4 were unable to survive at 37°C [31].

Under normal culture conditions, translation of *ZC3H11* mRNA is repressed. The 5'-UTR and coding sequences of the *ZC3H11* mRNA were not required, but a 71nt region within the 3'-UTR was necessary for stress-dependent translation repression. The activity of this sequence was context-dependent, since it did not work when placed next to the termination codon, or between two truncated (100nt) copies of the *ACT* 3'-UTR, but it was functional when inserted between two 625nt copies of fragment #1. The 71nt sequence may have to be located at a specific distance from the termination codon or poly(A) tail, or its secondary structure might be influenced by surrounding sequence. The regulatory element might be bound by a repressive RNA-binding protein. Preliminary attempts to identify such a protein through affinity purification with a streptavidin aptamer [32] failed. We have, however, already identified a number of RNA-binding proteins that are capable of repressing expression of a bound reporter mRNA in bloodstream forms [33,34], and we have a panel of bloodstream-or procyclic-form trypanosome lines containing inducible RNAi constructs. We tested the effects of inducing RNAi against DRBD2, DRBD7, PUF3, RBP9, RBP31, ZC3H8, ZC3H13, ZC3H22, ZC3H32, ZC3H35, ZC3H39, 4E-IP and Tb927.11.14220. None of these RNAi's resulted in an increase in ZC3H11 protein (S2C Fig), but this is inconclusive because the RNAi might not have reduced the target protein sufficiently to have an effect.

**Fig 11:**
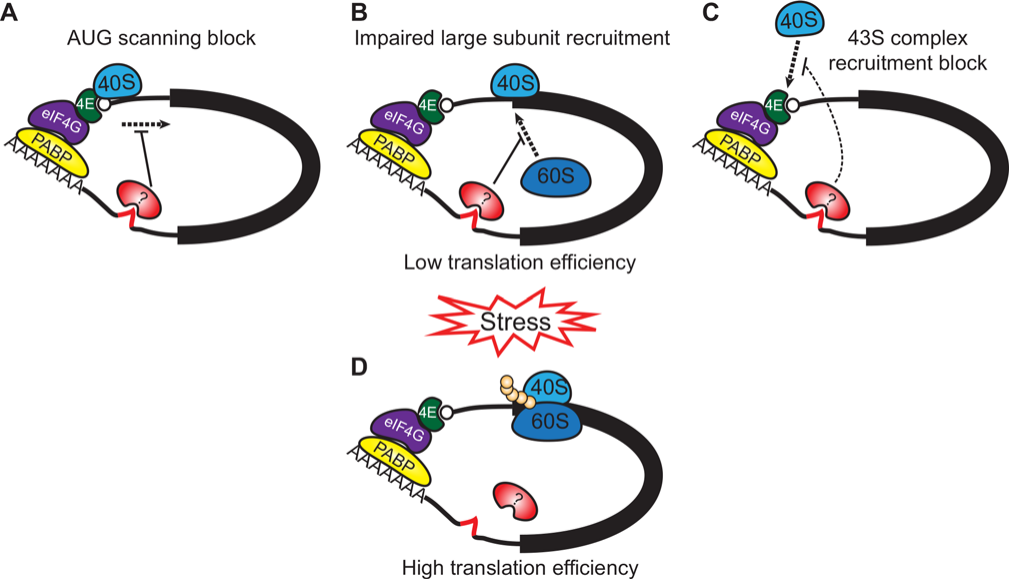
Proposed model of the translation regulation by the *ZC3H11* 3'-UTR. In unstressed cells the *ZC3H11* mRNA co-migrates with small ribosomal subunit suggesting repression of translation initiation via either a block of start codon scanning (A) or impaired large subunit recruitment (B). The alternative possibility is that initiation is halted at a step prior to 43S recruitment and the *ZC3H11* mRNA with its associated proteins is heavy enough to migrate in 40S fraction (C). A protein (?) bound to the 71nt sequence in the 3'-UTR is in some way responsible for these effects. Upon stress (heat shock, puromycin, MG132, arsenite) this protein is either destroyed, replaced by a different protein, or modified such that it no longer has suppressive activity. The *ZC3H11* mRNA then shifts to heavy polysomes (D).

Eukaryotic translation initiates when the 43S complex—which contains the 40S subunit, various translation factors, and charged tRNA—is recruited to the 5'-end of an mRNA by the eIF4E/G complex, making a 48S complex. The tRNA-ribosome-factor complex then scans to the initiation codon before large subunit joining [35]. There are three general mechanisms by which translation of *ZC3H11* mRNA may be inhibited. The first two require that the 48S complex is associated with the RNA, which initially seemed possible since the non-translated *ZC3H11* mRNA migrates at about 40S on sucrose gradients. Sequence-specific stalling of the subunit within the 5'-UTR (Fig 11A) can be ruled out, since neither the 5'-UTR nor the coding region was required for translational repression. An alternative would be that the 40S-subunit-containing complex is stalled at the start codon (Fig 11B). General translation inhibition by Reaper works this way [36], and sequence-specific control of the same type has been suggested for three mRNAs, although the evidence was somewhat circumstantial [37–39]. The most commonly described mechanism of sequence-specific translation regulation in eukaryotes is, however, a complete failure of 43S recruitment [35] (Fig 11C). We favour this option, because the ZC3H11 usually migrated at, but not below, 40 S, and we found no evidence for association of a 40-48S complex with any particular region of the mRNA (S3C Fig). Since a single RNA binding protein is likely to occupy between 4 and 20 residues [40–42], the 3.5kb *ZC3H11* mRNA could be associated with more than 50 RNA-binding proteins, resulting in migration at a similar position to ribosomal subunits.

Trypanosomes have several homologues of the cap-binding translation initiation complex components eIF4E and eIF4Gs [43–45]. In *T*. *brucei*, eIF4E1 represses expression when tethered to a reporter, as does its binding partner 4E-IP [33,34], but RNAi targeting these did not increase ZC3H11 expression (S2C Fig). We nevertheless speculate that under non-stressed conditions, a protein or proteins associated with the *ZC3H11* 3'-UTR recruits a translationally inactive 4E-containing complex to the *ZC3H11* 5' cap. Upon heat shock, normal eIF4E4/G3-dependent translation is shut down, but translation of the *ZC3H11* mRNA escapes, either through activation/modification of its existing cap-bound complex, or through exchange for a new, active one (Fig 11D).

Our experiments have revealed two mechanisms by which ZC3H11 levels are regulated: protein degradation and translation initiation. Neither form of control is well understood in kinetoplastids. Firstly, under normal conditions, ZC3H11 is rapidly degraded by the proteasome. Although the proteasome itself has been well characterized [46,47], and various proteins have been shown to be ubiquitinated prior to degradation [48–50], we know almost nothing about how the ubiquitination machinery recognizes appropriate targets. Secondly, *ZC3H11* mRNA translation is tightly controlled. Despite evidence for translational regulation of hundreds of mRNAs [3,4], we know little about what the different translation initiation complexes do or how they are regulated. Extensive further work in both these areas will be required in order to understand ZC3H11 regulation.

## Material and Methods

### Ethics statement

The antisera against ZC3H11 were generated by Charles River Laboratories, Kisslegg, Germany. The company is responsible for compliance with all relevant regulations regarding animal welfare.

### Trypanosome culturing and plasmids

Details of all plasmids and oligonucleotides are provided in S1 Table. Site-directed mutagenesis method was used to generate deletions and G→C mutations within the plasmids with wild-type *ZC3H11* 3'-UTR sequence. The culture conditions were as described in [51]. Procyclic trypanosomes were grown in MEM-Pros medium at 27°C (unless stated otherwise) at densities lower than 6×10^6^ cells/ml. Bloodstream forms were grown in HMI-9 medium. Nearly all experiments were done with Lister 427 monomorphic procyclic form parasites expressing the Tet-repressor, except one case (Fig 5B) where bloodstream forms were used. Stable cell lines were created with constitutive expression of *CAT* reporter mRNAs, or with sequences encoding the V5 or myc tag in frame with open reading frames of interest. Additional lines had tetracycline-inducible expression of dsRNA or tagged proteins. For these, expression was induced using 200ng/ml tetracycline. All plasmids used are listed in S1 Table.

### Recombinant ZC3H11 expression and antibody production

Fragments of the *ZC3H11* open reading frame (first 104a.a., 119a.a., 136a.a.) were cloned into pQEA38vector after His_10_-tag, between *KpnI* and *HindIII* sites. Bacteria (*E*.*coli* strain Rosetta pLysS, Novagen) were grown at room temperature to an OD600 of 0.6, induced with 0.25mM isopropyl β-D-1-thiogalactopyranoside and incubated at the same temperature for five hours before harvesting. Recombinant proteins were purified with Ni-NTA Agarose (QIAGEN) following the manufacturers’ instructions [20]. Buffer in protein samples was exchanged to PBS. Rabbits were immunized with His_10_-ZC3H11 (119a.a.) according to standard procedures (Charles River Laboratories, Kisslegg, Germany). Polyclonal antibodies were affinity-purified from crude anti-serum using His_10_-ZC3H11 (119a.a.) fragment coupled to CNBR-activated Sepharose (GE Healthcare).

### Trypanosome fractionation

Cytoskeleton-free extracts were obtained as previously described [52]. Cells were harvested by centrifugation (850g, 8min, 20°C), washed with cold phosphate-buffered saline and lysed in extraction buffer (1% (vol/vol) IGEPAL in 0.1M PIPES, 2mMEGTA, 1mM MgSO_4_, 0.1mM EDTA, pH6.9, supplemented with 10μg/ml leupeptin and tablet/10ml of PhosSTOP Phosphatase Inhibitor Cocktail, Roche). After centrifugation (3400g, 10min, 4°C), supernatant was taken and resuspended in 2× Laemmli buffer.

### Protein half-life measurement

1×10^8^ procyclic cells with or without a 1 hour heat shock were treated with 100μg/ml cycloheximide 5min prior to starting the indicated time course, and 1×10^7^ cells were collected at the indicated time points. The endogenous ZC3H11 protein was detected by Western blotting in cytoskeleton-free extracts using anti-ZC3H11 antibodies. Alternatively, cells were subjected to [^35^S]-methionine pulse labelling as described in [53,54]. The ZC3H11-myc protein expressed from modified endogenous ZC3H11 locus was detected in total lysate using anti-myc antibodies. Quantification was done using MultiGauge or Adobe Photoshop Software. A band that cross-reacted with the anti-ZC3H11 antibody, or Ponceau S staining, were used as loading controls.

### Protein detection and manipulation

Immunoprecipitation assays were done as previously described [21]. 1×10^8^ procyclic trypanosomes expressing V5-CK1.2 were harvested by centrifugation (850g, 8min, 20°C), washed with 1ml of cold phosphate-buffered saline and lysed in hypotonic buffer (10mM NaCl, 10mMTris-Cl pH7.5, 10μg/ml leupeptin,0.1%NP40)by passing 20–30 times through a 21G needle. After pelleting insoluble debris by centrifugation (17000g, 10min, 4°C) and adjusting to 150mM NaCl, the clarified lysate was used for immunoprecipitation with 200μl of anti-V5-coupled beads (Bethyl Laboratories) for 2 hours at 4°C. The beads were washed then 5 times at 4°C with IPP150 (10mM Tris pH7.5; 150mM NaCl; 0.1% IGEPAL) and divided into 4×50μl aliquots for *in vitro* kinase assay. Samples for western blots were taken during the procedure.

Proteins were detected by western blotting according to standard protocols. For detection of the endogenous ZC3H11 protein only cytoskeleton-free extracts were used. Antibodies used were to the ZC3H11 (rabbit, 1:10000, this paper), to V5 tag (AbD seroTec, 1:1000), the myc tag (Santa Cruz Laboratories, 1:1000), aldolase (rabbit, 1:50000[54]). Detection was done using ECL solutions (GE Healthcare). Chloramphenicol acetyltransferase activity was measured in a kinetic assay involving partition of ^14^C-buturyl chloramphenicol from the aqueous to the organic phase of scintillation fluid [55]. Total protein concentration was measured by Bradford method.

### Polysome fractionation

3–5×10^8^ procyclic cells were treated with cycloheximide (100μg/ml) for 5minutes, harvested at room temperature by centrifugation (850g, 8min, 20°C), washed once in 1ml of ice-cold PBS and lysed in 300μl of lysis buffer (20mM Tris pH7.5, 20mM KCl, 2mM MgCl_2_, 1mM DTT, 1200u RNasin (Promega), 10μg/ml leupeptin, 100μg/ml cycloheximide, 0.2% (vol/vol) IGEPAL) by passing 20–30 times through a 21G needle. After pelleting insoluble debris by centrifugation (17000g, 10min, 4°C) and adjusting to 120mM KCl, the clarified lysate was layered onto a 17.5–50% sucrose gradient (4ml) and centrifuged at 4°C for 2 hours at 40000 rpm in Beckman SW60 rotor. Monitoring of absorbance profiles at 254nm and gradients fractionation was done with a Teledyne Isco Foxy Jr. system. A human β-globin *in vitro* transcript was added to each of the collected fractions as a spike-in control.

### Northern blotting

Total RNA was extracted using peqGOLDTrifast (Peqlab). Isolated RNA (typically 20μg of total RNA or RNA purified from entire gradient fraction) was resolved on formaldehyde agarose gel, blotted to nylon membranes and detected by hybridization with radioactive probes for *CAT*, *ZC3H11*, *NPT1*, *HSP70* (Tb927.11.11330) and β-tubulin (Tb927.1.2370) mRNAs. Total mRNA was detected using an oligonucleotide antisense to mini-exon. Quantification was done using MultiGauge Software. Signal from 7SL RNA was used to measure loading.

### *In vitro* phosphorylation assays

Kinase assays using purified V5-CK1.2 were performed as described [56]. Phosphorylation reactions (10μl total volume) using the immunoprecipitated V5-CK1.2 contained 10μCi of [γ-^32^P]-ATP in1× NEBuffer for Protein Kinases (50mM Tris-HCl pH8.0, 10mM MgCl_2_, 0.1mM EDTA, 2mM DTT, 0.2% (vol/vol) IGEPAL) and 5μg of BSA (control) or recombinant His_10_-ZC3H11 N-terminal fragments (first 104, 119 or 136 amino acids). Reactions were allowed to proceed for 20min at room temperature. Proteins were resuspended in 2×Laemmli buffer, resolved by SDS-PAGE, stained with Coomassie blue and the incorporation of radioactive phosphate into recombinant ZC3H11 fragments was detected by autoradiography.

### RNase H cleavage

RNase H cleavage was done in clarified lysate from 4×10^8^ procyclic cells obtained in the same way as for polysome fractionation. Lysate was divided into two portions (~200μl each) and mix of two oligonucleotides antisense to the *ZC3H11* mRNA was added to the final concentration 2μM. One oligonucleotide annealed 81 nucleotides upstream the start codon (cz5636), second annealed 295 nucleotides downstream the start codon (cz4596). Both tubes were incubated at 37°C with slow cooling to room temperature during 20min. 10u of RNase H (Thermo Fisher Scientific) were added to one of the tubes and incubated for another 20min at 37°C. Then both control and RNase H treated lysates were layered onto a 10–30% sucrose gradient (4ml) and centrifuged at 4°C for 2 hours at 40000 rpm in Beckman SW60 rotor. RNA isolated from 15 collected fractions was probed with 5'-end radiolabeled oligonucleotides antisense to *ZC3H11* mRNA in order to detect three fragments of cleavage: 5'-UTR fragment (216nt + splice leader, probe cz5635), 5'-ORF fragment (372nt, probe cz5891) and 3'-ORF/3'UTR fragment (2314nt + poly(A) tail, cz5824).

### Purification of trypanosome heat shock granules

Granules from normal and heat-shocked procyclic cells were enriched as described previously [28]. 5×10^8^ control or heat-shocked (1 hour at 41°C) procyclic cells were harvested at room temperature by centrifugation (1500g, 10min), washed in 1ml of PBS and lysed in 200μl of ice-cold buffer A (20mM Tris-HCl pH 7.6, 2mM MgCl_2_; 0.25M sucrose, 1mM DTT, 10% glycerol, 1% TritonX100, 800u RNasin (Promega), 1 tablet Complete Protease InhibitorCocktail EDTA free (Roche)/10ml buffer) by pipetting. Lysis was confirmed microscopically. The lysate was clarified (20000g, 10min) and the supernatant (SN1) was transferred to fresh tube with 750μl of peqGOLDTrifast FL(Peqlab). All remaining supernatant was removed after one short centrifugation (3min, 20.000g). The pellet was resuspended again in 200μl of buffer A by passing 30–40 times through a 21G syringe, vortexed and centrifuged (20000g, 5min). The supernatant (SN2) was taken and the pellet was resuspended in 200μl buffer A as above. Whole procedure was repeated one more time to obtain the supernatant SN3. Then the pellet was resuspended one more time in 200μl buffer A as above and microtubules were disrupted by the addition of 12 μl 5M NaCl (283mM final conc.), the samples were passed through 21G syringe, incubated on ice for 30 minutes with vortexing every 5 minutes and centrifuged (20000g, 10min). The supernatant (SN4) was removed up and the pellet was washed once in 200μl of buffer A without resuspension (20000g, 10min) and finally resuspended in 750μl of Trifast FL. Another 5×10^7^ control or heat-shocked procyclic cells were taken to obtain total RNA.

### mRNA FISH and microscopy

5×10^7^ procyclic cells (control or heat-shocked for 1 hour at 41°C) expressing CAT reporter with full-length or fragment #1 (1-625nt) ZC3H11 3'-UTR were washed in PBS, pelleted (1400g, 10min), resuspended in 1ml PBS, fixed by the addition of 1ml 8% paraformaldehyde in PBS for 10min and pelleted again after the addition of 13ml PBS. The cells were resuspended in 1ml PBS and allowed to settle on a baked superfrost microscopy slide (within hydrophobic circles) for 15min. Affymetrix FISH was done with the QuantiGene ViewRNA ISH Cell Assay kit (Affymetrix), according to the manufacturer's instructions, but with protease digestion for 30min at the highest suggested concentration (1:500)[28]. This treatment increases the number of visualised mRNA molecules, but also causes cell loss and disrupted cell morphology [28]. The CAT ORF Affymetrix probe sets were used in a 1:100 dilution of the original stock and DNA was stained with the DAPI.Z-stack images (100 stacks at 100nm distance) were taken with a custom build TILL Photonics iMic microscope equipped with a sensicam camera (PCO), deconvolved using Huygens Essential software and are, unless otherwise stated, presented as Z-projections (method sumsliced) produced by ImageJ software.

## Supporting Information

S1 Table Plasmids and oligonucleotides used in this paper. (XLS)

S1 Fig Anti-ZC3H11 antibody specificity and ZC3H11 protein amount in procyclics. **A.** We immunized a rabbit with the N-terminal 119 amino-acids of ZC3H11 (produced as a His-tagged protein in E. coli) and affinity purified the resulting polyclonal antibodies. Serial dilutions of the recombinant protein were resolved on SDS-PAGE and probed with the Ab at 1:10000 dilution. The antibodies had a detection limit of about 200pg of the recombinant polypeptide. **B.** The specificity of anti-ZC3H11 antibodies was tested on total and cytoskeleton-free trypanosome samples. Procyclic trypanosomes with or without RNAi against ZC3H11 were heat-shocked at 37°C for 1 hour and extracts from 5×10^6^ cells were loaded per lane. With total lysate (lanes 1–4) the antibodies showed multiple bands, with no evidence for any specific recognition of ZC3H11. There was a very strong signal at about 50 kDa—the same position as alpha and beta tubulin. Cytoskeleton-free extracts (lanes 5–8) were therefore obtained as described in Materials and Methods. The putative ZC3H11 protein band is indicated with an arrow (lane 6). This is present only in heat-shocked cells without RNAi. A cross-reacting band (probably residual tubulin) is indicated by an asterisk. After more careful fractionation this band is usually not seen, as judged by signals at 27°C (Fig 1A) and controls with RNAi (Fig 2A, lane 15). **C.** Pull-down with anti-ZC3H11 antibodies. Extracts from 5×10^7^ procyclic cells ectopically expressing full-length or N-terminal fragment of myc-tagged ZC3H11 were subjected to immunoprecipitation with anti-ZC3H11. The efficiency of immunoprecipitation was analysed by Western blotting using anti-myc. In: input, U: unbound (2×10^6^ cell-equivalents), E: eluate (5×10^6^ cell-equivalents). **D.** Western blot analysis of ZC3H11-myc obtained by cell fractionation. Cytoskeleton-free extracts from control and heat-shocked procyclic cells ectopically-expressing ZC3H11-myc protein were analyzed by immunoblot with anti-myc and, as control, anti-aldolase. Approximately 90% of ectopically-expressed ZC3H11-myc protein remained in the supernatant after cytoskeleton depletion (S1D Fig). **E.** Expression of endogenous ZC3H11 in procyclic cells after heat shock. Cells were treated for 1 hour with mild (37°C) or severe (41°C) heat shock. To the cytoskeleton-free extracts from 5×10^6^ cells were added dilutions of recombinant ZC3H11 fragment and analyzed by immunoblotting. Detection was with anti-ZC3H11 and cross- reacting band is shown as loading control. One representative image from three separate experiments is shown. **F.** ZC3H11 protein levels in procyclic cells with normal and heat shock conditions. Quantification from three replica immunoblots is shown. We cannot exclude some cross-reactivity in the signal for 27°C. **G.** Dynamics of ZC3H11 expression in procyclic forms during mild heat shock and a recovery period. The cells were incubated for 1h at 37°C, and then transferred to normal conditions. The loading control is one of the major cross-reacting bands seen in panel B. ZC3H11 accumulation was visible within 5 minutes. Loss of ZC3H11 at 27°C was slower: it started to decrease only after an hour, but was notably less abundant after 5h.
(PDF)

S2 Fig Negative results. **A.** Stresses that do not induce ZC3H11 expression in procyclic forms. All treatments were for 1h and ZC3H11 was detected in cytoskeleton-free extracts using the polyclonal antibody. A cross-reacting band served as a loading control. **B.** RNAi targeting protein kinases Tb927.10.5140 and Tb927.5.2820 has no effect on ZC3H11 band migration. RNAi was induced for 2 days, then the cells were transferred to either 37°C or 41°C for 1 hour. ZC3H11 was detected by western blotting, with a cross-reacting band that is unaffected by heat shock as loading control. Cytoskeleton-free extracts from 5×10^6^ cells were loaded on each lane. One representative image from three separate experiments is shown. **C.** Effect of RNAi targeting DRBD2, DRBD7, PUF3, RBP9, RBP31, ZC3H8, ZC3H13, ZC3H22, ZC3H32, ZC3H35, ZC3H39, 4E-IP and Tb927.11.14220 on ZC3H11 expression in bloodstream and procyclic trypanosomes. Procyclic RNAi cell lines targeting DRBD2, ZC3H39, ZC3H22, 4E-IP and bloodstream form RNAi cell lines targeting DRBD7, PUF3, RBP9, RBP31, ZC3H8, ZC3H13, ZC3H32, ZC3H35, Tb927.11.14220 were induced with tetracycline (200g/ml) for 2 days. The unstressed and heat-shocked parental cell lines were used as a control. ZC3H11 levels were analysed by Western blotting. 5×10^6^ cells were loaded per lane. A cross-reacting band served as a loading control.
(PDF)

S3 Fig **A. *ZC3H11* mRNA migrates near the 40S peak in 10–30% sucrose gradients.** The upper panel shows absorbance at 254nm after 10–30% sucrose density gradient centrifugation of extracts from procyclic trypanosomes grown at 27°C. Peaks for small (40S), monosome (80S) and polysomes are indicated with arrows. The lower panels show the corresponding methylene blue stain (for rRNA) and Northern blot detection of *ZC3H11* mRNA. **B.** Cycloheximide treatment does not affect *ZC3H11* mRNA migration in sucrose gradients. Extracts from untreated or cycloheximide treated procyclic cultures grown at 27°C were subjected to 17.5–50% sucrose gradient centrifugation. The upper panels show representative absorbance profiles at 254 nm, and the lower panels are Northern blots of RNA preparations from the different fractions. A methylene blue stain is shown beneath the fraction numbers. *ZC3H11* mRNA was detected in both blots. **C.** Cutting of *ZC3H11* mRNA with RNase H moves the fragments into a fraction above the 40S peak. Procyclic trypanosome extracts suitable for polysome analysis were treated with RNase H in the presence of oligonucleotides targeting the *ZC3H11*mRNA (bottom panel). Antisense oligo R1 annealed 81 nucleotides upstream the start codon and R2 (black arrows) annealed 295 nucleotides downstream. The extracts were then fractionated on 10–30% sucrose gradients, with untreated extracts as the control. The top panel shows representative 254nm absorbance profiles. Peaks for small (40S), large (60S) ribosomal subunit and monosome (80S) are indicated with arrows.The central panels show Northern blot detection of full-length and cleavage products of the *ZC3H11* mRNA. rRNA staining with methylene blue is shown above. The lines on the absorbance profile plot show the times at which the fractions shifted, which do not correspond exactly to the tube contents. The cleavage products were detected with 5'-end radiolabeled antisense oligonucleotides P1-3 (red arrows): 5'-UTR fragment (Frag.1: 216nt + splice leader), 5'-ORF fragment (Frag. 2: 372nt) and 3'-ORF/3'-UTR fragment (Frag. 3: 2314nt + poly(A) tail). If a 40S subunit were arrested at the start codon, we would expect Fragment 2 to migrate at 40S; if it were stuck at the cap we would expect Fragment 1 to migrate at 40S. If the migration were due to association with RNA-binding proteins, we would expect the much longer Fragment 3—which contains most of the open reading frame, and the 3'-UTR and poly(A) tail—to migrate at 40S, unless proteins are bound much more densely to the 5'-UTR and cap. In fact all of the fragments now migrated slightly lighter than 40S.
(PDF)

S4 Fig Deletion of nt 945–982 and 1003–1076 do not affect regulation by the *ZC3H11* 3'-UTR. **A.** Relative CAT mRNA and CAT activity levels, with or without 24h at 37°C, were determined by Northern blotting and CAT assay (shown as mean ± standard deviation, n = 3). **B.** Representative absorbance profiling at 254nm obtained by sucrose density gradient centrifugation for procyclic cultures before and after one hour at 37°C. Peaks for small (40S), large (60S) ribosomal subunit, monosome (80S) and polysomes are indicated with arrows. **C.** Quality and distribution of the CAT mRNA across the sucrose gradient fractions analyzed by Northern blotting (NB). In vitro transcribed human β-globin RNA was added as a spike-in control of equal RNA isolation efficiency. Results from one representative experiment out of two are shown. Results for WT fragment #2, the ∆894–924 mutant and methylene blue staining, are from Fig 8.
(PDF)

S5 Fig Prediction of the structures of wild-type and mutated fragment #2 of *ZC3H11* 3'-UTR. **A.** Prediction by Mfold for the complete fragment, and various versions with deletions of predicted loops. The targeted stem-loops are highlighted with green, and the 894–924 segment is shown on a larger scale. **B.** Predicted effects of deletion of a 13nt GU-rich sequence, and G-to-C exchange at positions 908–920 on the secondary structure of fragment 2 of *ZC3H11* 3'-UTR. Mutated nucleotides are indicated in red.
(PDF)
