# Supplementary material for: Regulating a Post-Transcriptional Regulator: Protein Phosphorylation, Degradation and Translational Blockage in Control of the Trypanosome Stress-Response RNA-Binding Protein ZC3H11
Source: PLoS Pathog. 2016 Mar 22;12(3):e1005514. doi: 10.1371/journal.ppat.1005514 (PMC4803223; doi:10.1371/journal.ppat.1005514)
Supplement: S1 Fig — A. We immunized a rabbit with the N-terminal 119 amino-acids of ZC3H11 (produced as a His-tagged protein in E. coli) and affinity purified the resulting polyclonal antibodies. Serial dilutions of the recombinant protein were resolved on SDS-PAGE and probed with the Ab at 1:10000 dilution. The antibodies had a detection limit of about 200pg of the recombinant polypeptide. B. The specificity of anti-ZC3H11 antibodies was tested on total and cytoskeleton-free trypanosome samples. Procyclic trypanosomes with or without RNAi against ZC3H11 were heat-shocked at 37°C for 1 hour and extracts from 5×10^6^ cells were loaded per lane. With total lysate (lanes 1–4) the antibodies showed multiple bands, with no evidence for any specific recognition of ZC3H11. There was a very strong signal at about 50 kDa—the same position as alpha and beta tubulin. Cytoskeleton-free extracts (lanes 5–8) were therefore obtained as described in Materials and Methods. The putative ZC3H11 protein band is indicated with an arrow (lane 6). This is present only in heat-shocked cells without RNAi. A cross-reacting band (probably residual tubulin) is indicated by an asterisk. After more careful fractionation this band is usually not seen, as judged by signals at 27°C (Fig 1A) and controls with RNAi (Fig 2A, lane 15). C. Pull-down with anti-ZC3H11 antibodies. Extracts from 5×10^7^ procyclic cells ectopically expressing full-length or N-terminal fragment of myc-tagged ZC3H11 were subjected to immunoprecipitation with anti-ZC3H11. The efficiency of immunoprecipitation was analysed by Western blotting using anti-myc. In: input, U: unbound (2×10^6^ cell-equivalents), E: eluate (5×10^6^ cell-equivalents). D. Western blot analysis of ZC3H11-myc obtained by cell fractionation. Cytoskeleton-free extracts from control and heat-shocked procyclic cells ectopically-expressing ZC3H11-myc protein were analyzed by immunoblot with anti-myc and, as control, anti-aldolase. Approximately 90% of ectopically-expressed ZC3H11-myc pro [file ppat.1005514.s002.pdf]

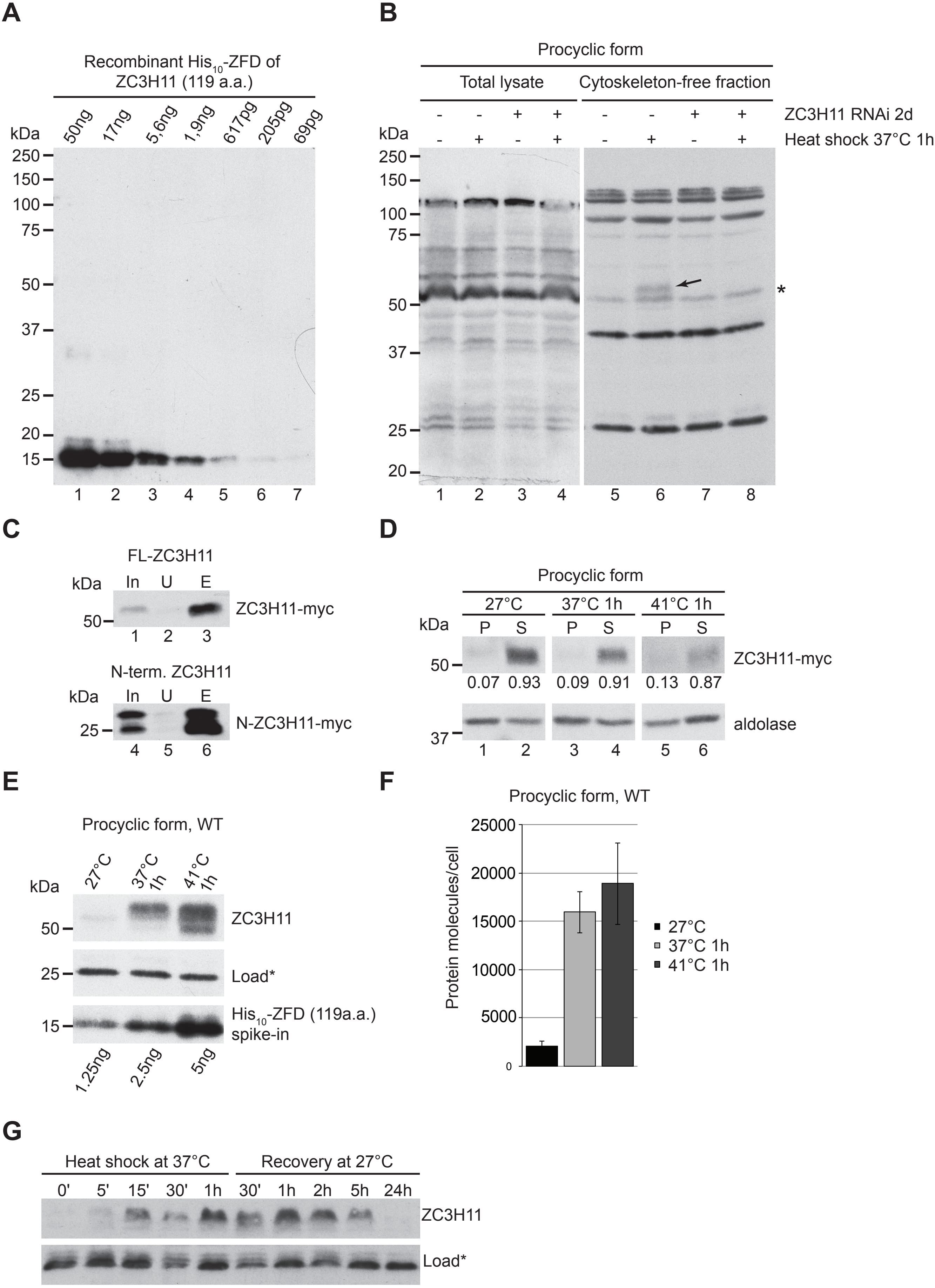
A
Recombinant His10-ZFD of ZC3H11 (119 a.a.)
50ng
17ng
5,6ng
1,9ng
617pg
205pg
69pg
kDa
250
150
100
75
50
37
25
20
15
1 2 3 4 5 6 7
B
Procyclic form
Total lysate
Cytoskeleton-free fraction
ZC3H11 RNAi 2d
Heat shock 37°C 1h
kDa
250
150
100
75
50
37
25
20
*
1 2 3 4 5 6 7 8
C
FL-ZC3H11
In U E
ZC3H11-myc
1 2 3
N-term. ZC3H11
In U E
N-ZC3H11-myc
4 5 6
D
Procyclic form
27°C
37°C 1h
41°C 1h
P S
ZC3H11-myc
0.07 0.93
0.09 0.91
0.13 0.87
aldolase
1 2 3 4 5 6
E
Procyclic form, WT
27°C
37°C 1h
41°C 1h
ZC3H11
Load*
His10-ZFD (119a.a.) spike-in
1.25ng
2.5ng
5ng
F
Procyclic form, WT
Protein molecules/cell
25000
20000
15000
10000
5000
0
27°C
37°C 1h
41°C 1h
G
Heat shock at 37°C
Recovery at 27°C
0' 5' 15' 30' 1h 30' 1h 2h 5h 24h
ZC3H11
Load*
