# Supplementary figures and images for: Regulating a Post-Transcriptional Regulator: Protein Phosphorylation, Degradation and Translational Blockage in Control of the Trypanosome Stress-Response RNA-Binding Protein ZC3H11
Source: PLoS Pathog. 2016 Mar 22;12(3):e1005514. doi: 10.1371/journal.ppat.1005514 (PMC4803223; doi:10.1371/journal.ppat.1005514)

**A**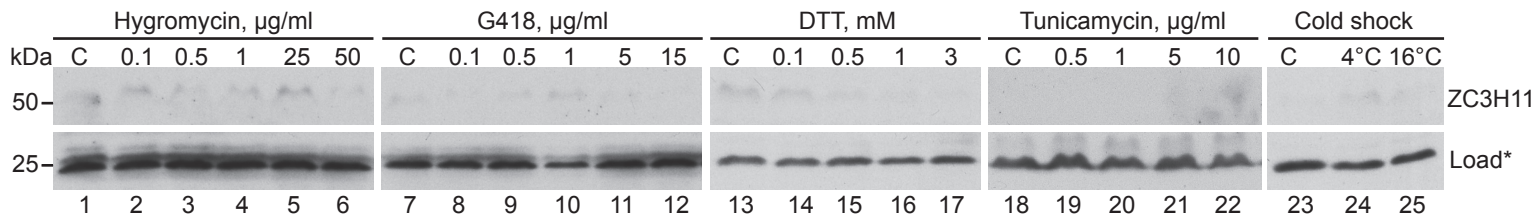**B**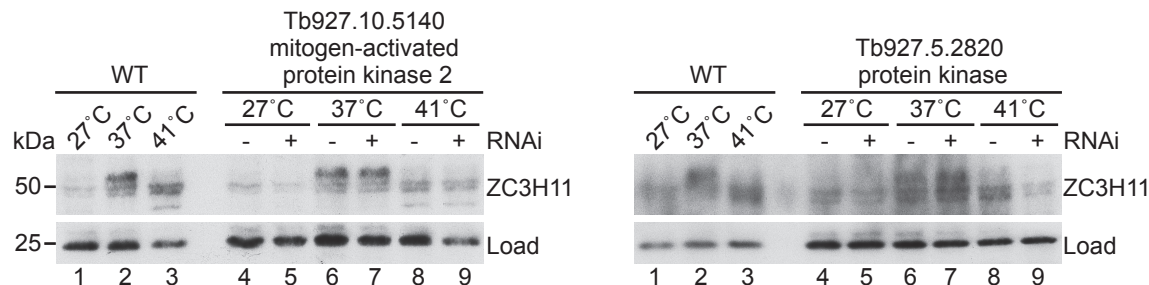**C**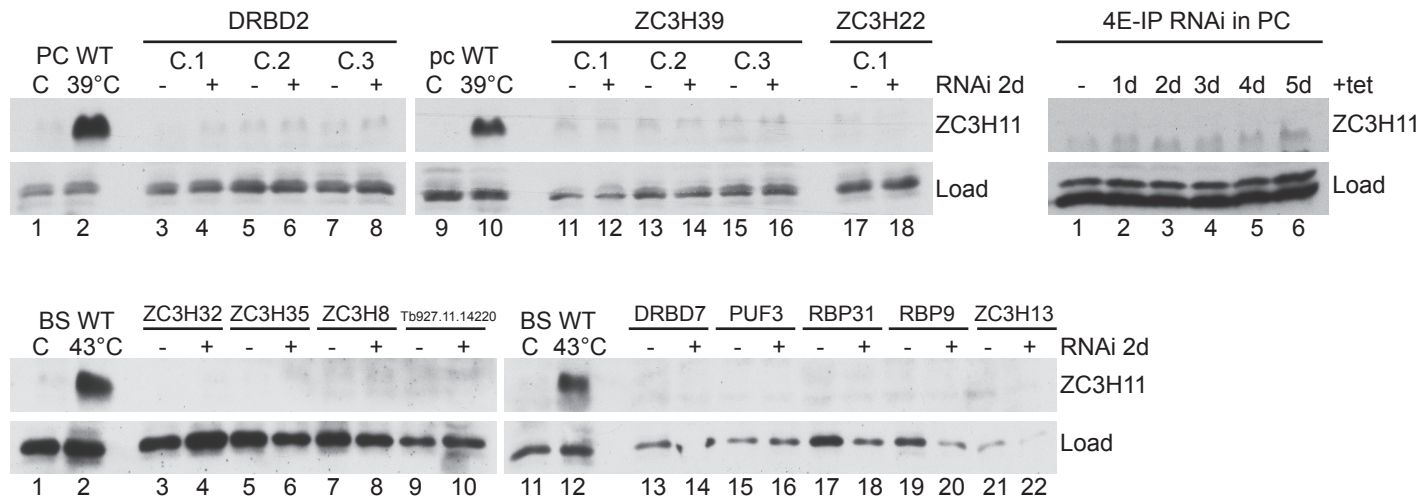

Supplement: S2 Fig — A. Stresses that do not induce ZC3H11 expression in procyclic forms. All treatments were for 1h and ZC3H11 was detected in cytoskeleton-free extracts using the polyclonal antibody. A cross-reacting band served as a loading control. B. RNAi targeting protein kinases Tb927.10.5140 and Tb927.5.2820 has no effect on ZC3H11 band migration. RNAi was induced for 2 days, then the cells were transferred to either 37°C or 41°C for 1 hour. ZC3H11 was detected by western blotting, with a cross-reacting band that is unaffected by heat shock as loading control. Cytoskeleton-free extracts from 5×10^6^ cells were loaded on each lane. One representative image from three separate experiments is shown. C. Effect of RNAi targeting DRBD2, DRBD7, PUF3, RBP9, RBP31, ZC3H8, ZC3H13, ZC3H22, ZC3H32, ZC3H35, ZC3H39, 4E-IP and Tb927.11.14220 on ZC3H11 expression in bloodstream and procyclic trypanosomes. Procyclic RNAi cell lines targeting DRBD2, ZC3H39, ZC3H22, 4E-IP and bloodstream form RNAi cell lines targeting DRBD7, PUF3, RBP9, RBP31, ZC3H8, ZC3H13, ZC3H32, ZC3H35, Tb927.11.14220 were induced with tetracycline (200g/ml) for 2 days. The unstressed and heat-shocked parental cell lines were used as a control. ZC3H11 levels were analysed by Western blotting. 5×10^6^ cells were loaded per lane. A cross-reacting band served as a loading control. (PDF) [file ppat.1005514.s003.pdf]

**A**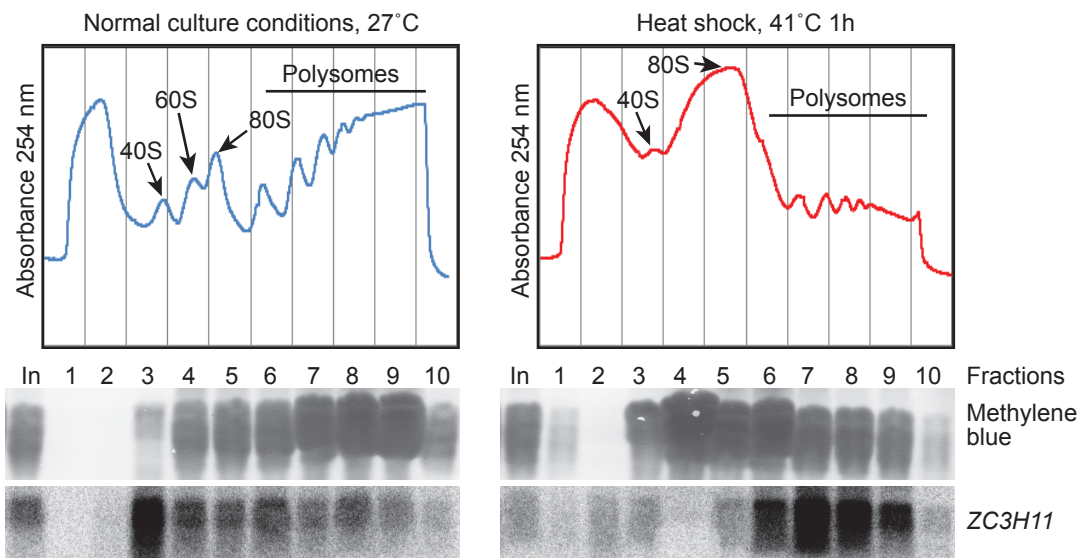**B**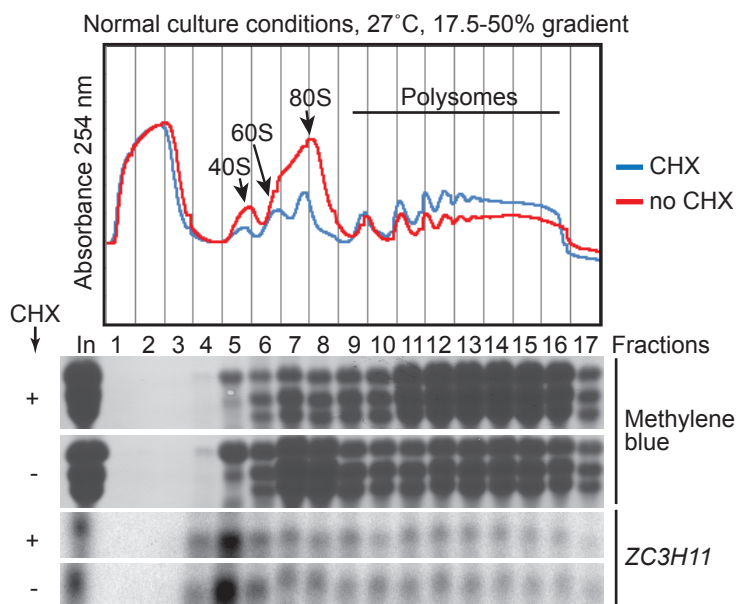**C**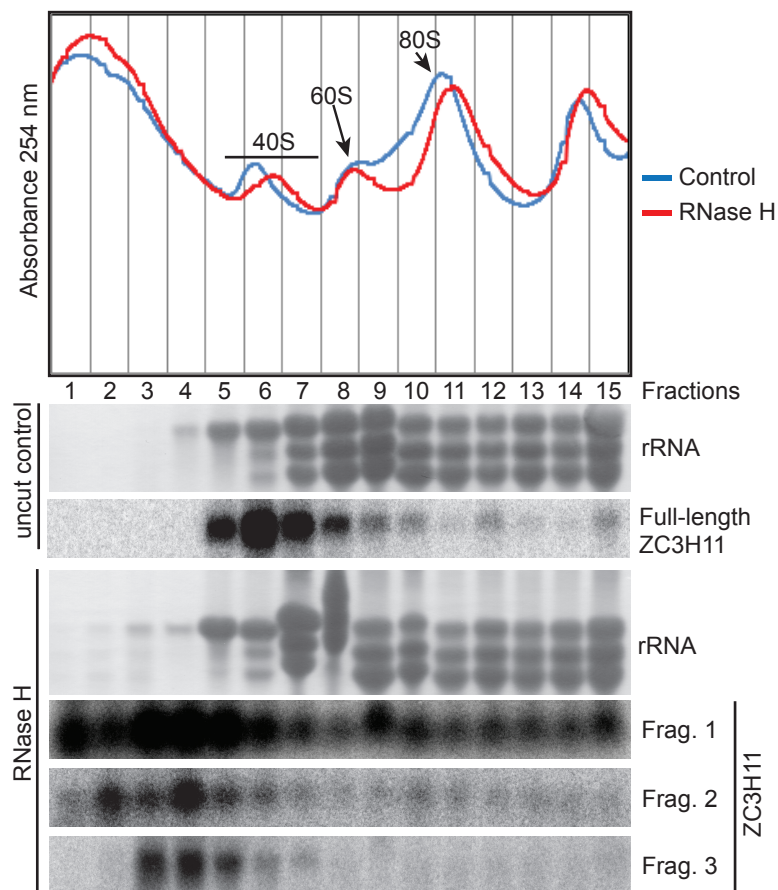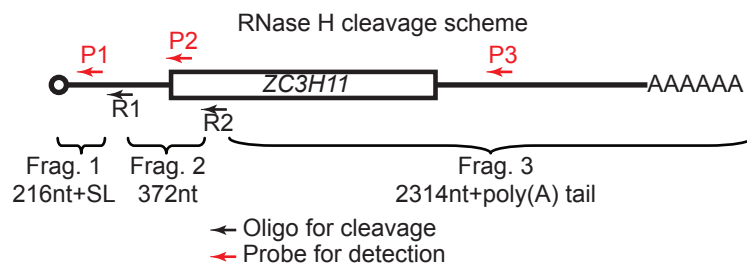

Supplement: S3 Fig — A. ZC3H11 mRNA migrates near the 40S peak in 10–30% sucrose gradients. The upper panel shows absorbance at 254nm after 10–30% sucrose density gradient centrifugation of extracts from procyclic trypanosomes grown at 27°C. Peaks for small (40S), monosome (80S) and polysomes are indicated with arrows. The lower panels show the corresponding methylene blue stain (for rRNA) and Northern blot detection of ZC3H11 mRNA. B. Cycloheximide treatment does not affect ZC3H11 mRNA migration in sucrose gradients. Extracts from untreated or cycloheximide treated procyclic cultures grown at 27°C were subjected to 17.5–50% sucrose gradient centrifugation. The upper panels show representative absorbance profiles at 254 nm, and the lower panels are Northern blots of RNA preparations from the different fractions. A methylene blue stain is shown beneath the fraction numbers. ZC3H11 mRNA was detected in both blots. C. Cutting of ZC3H11 mRNA with RNase H moves the fragments into a fraction above the 40S peak. Procyclic trypanosome extracts suitable for polysome analysis were treated with RNase H in the presence of oligonucleotides targeting the ZC3H11mRNA (bottom panel). Antisense oligo R1 annealed 81 nucleotides upstream the start codon and R2 (black arrows) annealed 295 nucleotides downstream. The extracts were then fractionated on 10–30% sucrose gradients, with untreated extracts as the control. The top panel shows representative 254nm absorbance profiles. Peaks for small (40S), large (60S) ribosomal subunit and monosome (80S) are indicated with arrows.The central panels show Northern blot detection of full-length and cleavage products of the ZC3H11 mRNA. rRNA staining with methylene blue is shown above. The lines on the absorbance profile plot show the times at which the fractions shifted, which do not correspond exactly to the tube contents. The cleavage products were detected with 5'-end radiolabeled antisense oligonucleotides P1-3 (red arrows): 5'-UTR fragment (Frag.1: 216nt + splice [file ppat.1005514.s004.pdf]

**A**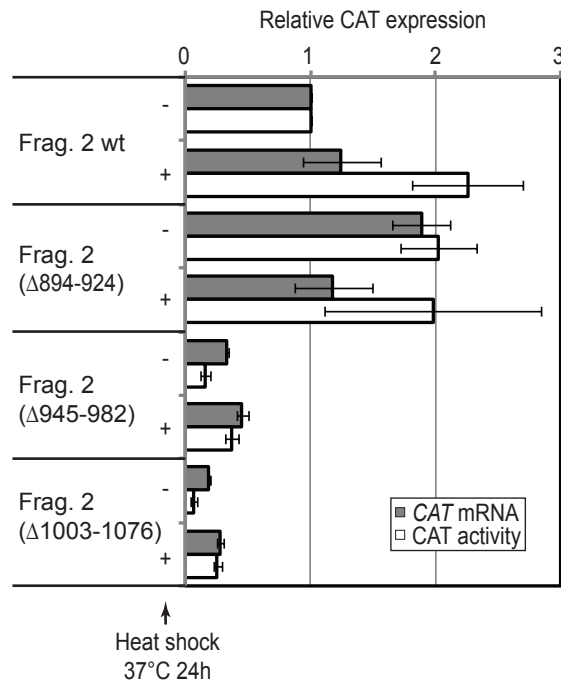**B**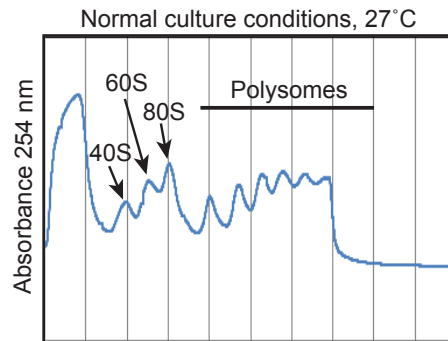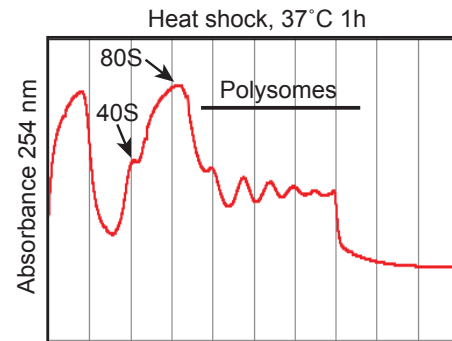**C**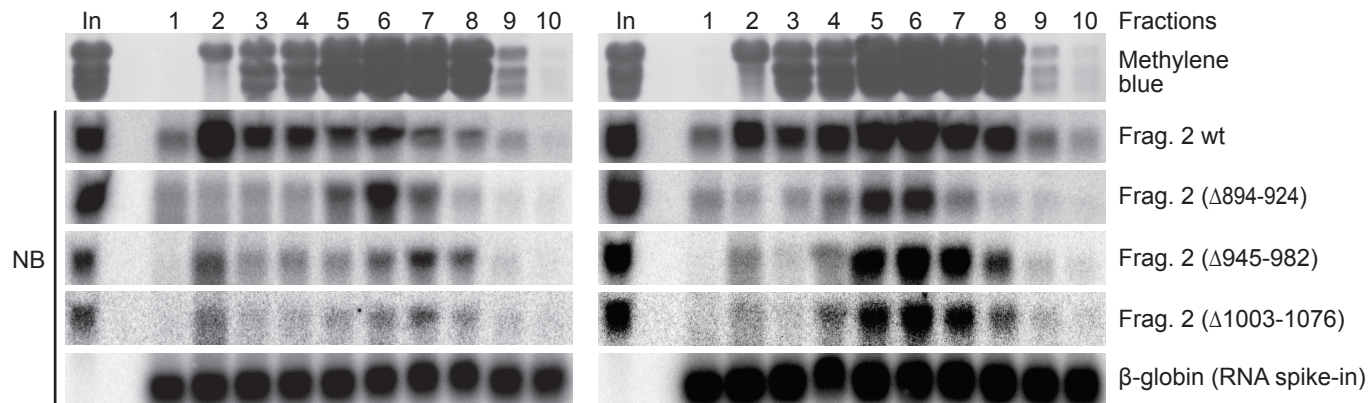

Supplement: S4 Fig — A. Relative CAT mRNA and CAT activity levels, with or without 24h at 37°C, were determined by Northern blotting and CAT assay (shown as mean ± standard deviation, n = 3). B. Representative absorbance profiling at 254nm obtained by sucrose density gradient centrifugation for procyclic cultures before and after one hour at 37°C. Peaks for small (40S), large (60S) ribosomal subunit, monosome (80S) and polysomes are indicated with arrows. C. Quality and distribution of the CAT mRNA across the sucrose gradient fractions analyzed by Northern blotting (NB). In vitro transcribed human β-globin RNA was added as a spike-in control of equal RNA isolation efficiency. Results from one representative experiment out of two are shown. Results for WT fragment #2, the ∆894–924 mutant and methylene blue staining, are from Fig 8. (PDF) [file ppat.1005514.s005.pdf]

**A**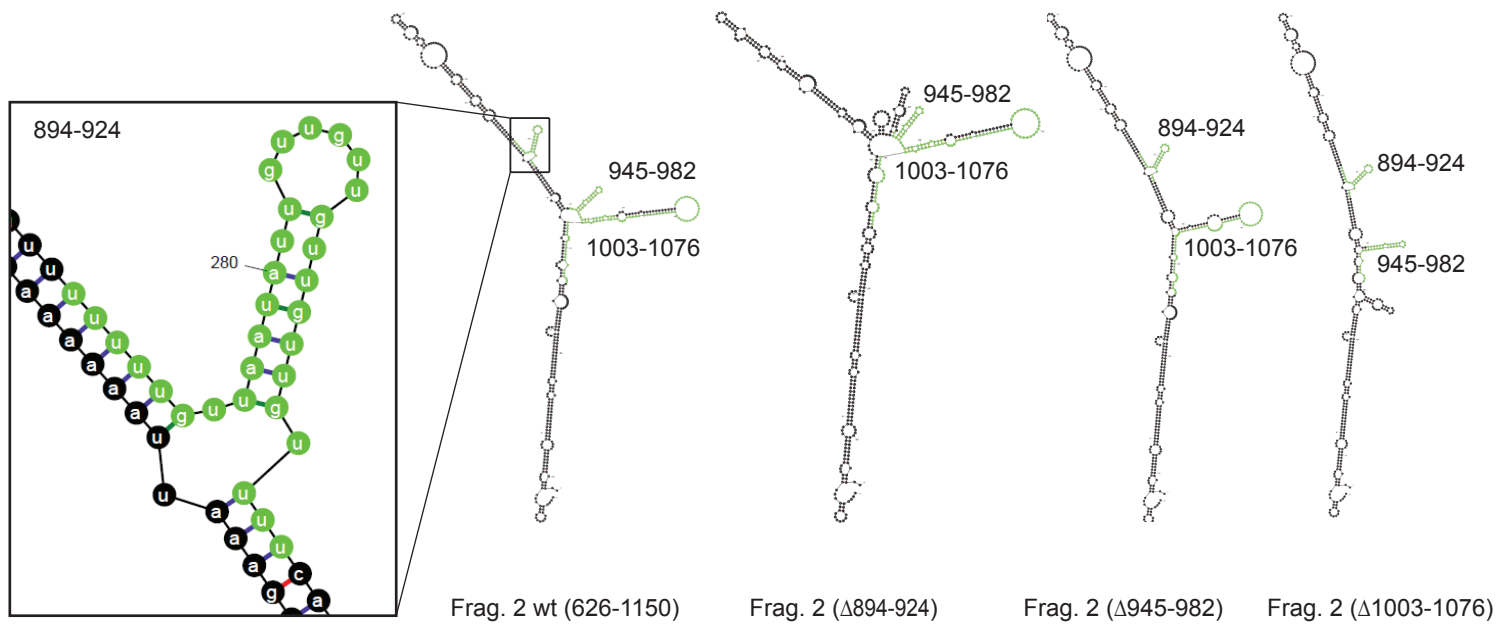**B**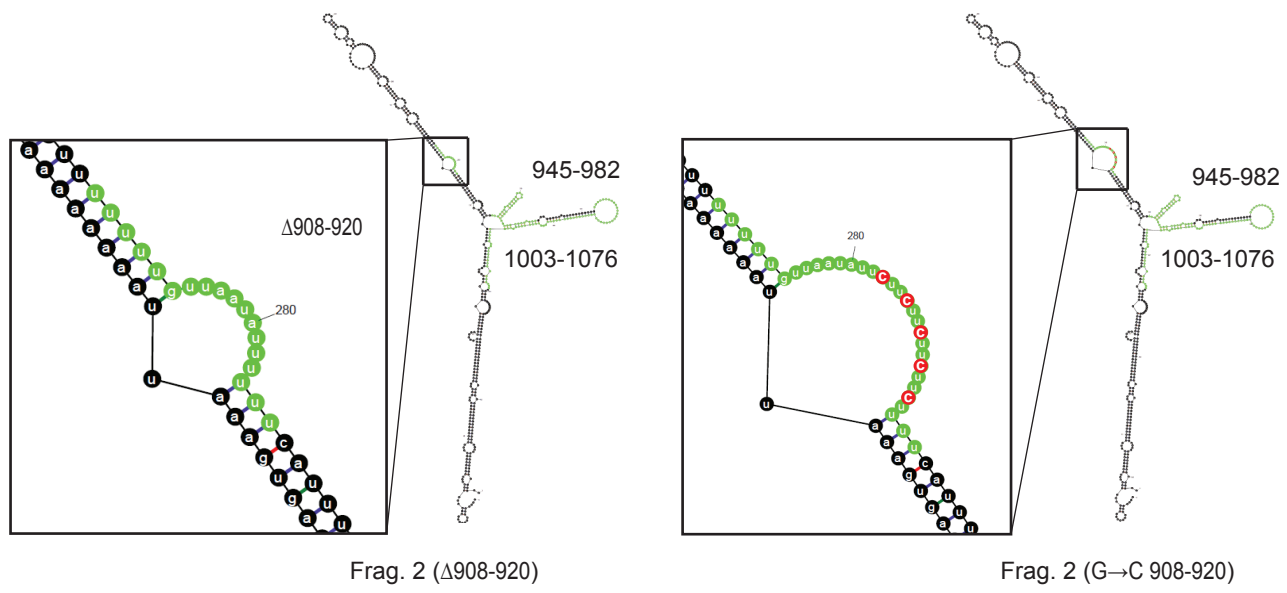

Supplement: S5 Fig — A. Prediction by Mfold for the complete fragment, and various versions with deletions of predicted loops. The targeted stem-loops are highlighted with green, and the 894–924 segment is shown on a larger scale. B. Predicted effects of deletion of a 13nt GU-rich sequence, and G-to-C exchange at positions 908–920 on the secondary structure of fragment 2 of ZC3H11 3'-UTR. Mutated nucleotides are indicated in red. (PDF) [file ppat.1005514.s006.pdf]
